# Variable Secondary Metabolite Profiles Across Cultivars of *Curcuma longa* L. and *C. aromatica* Salisb.

**DOI:** 10.3389/fphar.2021.659546

**Published:** 2021-06-30

**Authors:** Poonam Kulyal, Satyabrata Acharya, Aditya B. Ankari, Praveen K. Kokkiripati, Sarada D. Tetali, Agepati S. Raghavendra

**Affiliations:** Department of Plant Sciences, School of Life Sciences, University of Hyderabad, Hyderabad, India

**Keywords:** *Curcuma longa* L., *Curcuma aromatica* Salisb, essential oil, metabolomics, secondary metabolites, GC-MS, LC-MS

## Abstract

**Background:** *Curcuma* spp. (Zingiberaceae) are used as a spice and coloring agent. Their rhizomes and essential oils are known for medicinal properties, besides their use in the flavoring and cosmetic industry. Most of these biological activities were attributed to volatile and nonvolatile secondary metabolites present in the rhizomes of *Curcuma* spp. The metabolite variations among the species and even cultivars need to be established for optimized use of *Curcuma* spp.

**Objectives:** We compared the phytochemical profiles of rhizomes and their essential oils to establish the variability among seven cultivars: five of *Curcuma longa* L*.* (Alleppey Supreme, Duggirala Red, Prathibha, Salem, Suguna) and two of *C. aromatica* Salisb. (Kasturi Araku, Kasturi Avidi). The GC-MS and LC-MS-based analyses were employed to profile secondary metabolites of these selected cultivars.

**Methods:** Rhizomes of *Curcuma* spp. were subjected to hydro-distillation to collect essential oil and analyzed by GC-MS. The methanol extracts of fresh rhizomes were subjected to LC-MS analyses. The compounds were identified by using the relevant MS library databases as many compounds as possible.

**Results:** The essential oil content of the cultivars was in the range of 0.74–1.62%. Several compounds were detected from the essential oils and rhizome extracts by GC-MS and LC-MS, respectively. Of these, 28 compounds (13 from GCMS and 15 from LCMS) were common in all seven cultivars, e.g., α-thujene, and diarylheptanoids like curcumin. Furthermore, a total of 39 new compounds were identified from *C. longa* L. and/or *C. aromatica* Salisb., most of them being cultivar-specific. Of these compounds, 35 were detected by GC-MS analyses of essential oils, 1,2-cyclohexanediol, 1-methyl-4-(1-methylethyl)-, and santolina alcohol, to name a few. The other four compounds were detected by LC-MS of the methanolic extracts of the rhizomes, e.g., kaempferol-3,7-O-dimethyl ether and 5,7,8-trihydroxy-2′,5′-dimethoxy-3′,4′-methylene dioxyisoflavanone.

**Conclusions:** We identified and recorded the variability in the metabolite profiles of essential oils and whole rhizome extracts from the seven cultivars of *Curcuma longa* L. and *C. aromatica* Salisb. As many as 39 new metabolites were detected in these seven Indian cultivars of *Curcuma* spp. Many of these compounds have health benefits.

## Introduction

Turmeric (*Curcuma longa* L.) is a perennial rhizomatous herb that belongs to the family Zingiberaceae (Prasath et al., 2018). It has been used traditionally in India for its medicinal value and as a spice (Srinivasan et al., 2004; Aggarwal et al., 2007; Esatbeyoglu et al., 2012). In Ayurvedic medicine, turmeric is used internally (as a stomachic, tonic, and blood purifier) or externally (prevention and treatment of skin diseases) (Gounder and Lingamallu, 2012). Turmeric was scientifically validated for several pharmacological benefits, including antioxidant, anti-inflammatory, and chemoprotective properties (Miquel et al., 2002; Krup et al., 2013; Kanase and Khan, 2018; Umar et al., 2020). The rhizomes of turmeric are enriched with several bioactive metabolites, though the attention was mostly on curcuminoids. Besides curcumin (a curcuminoid), the essential oil of *C. longa* L. showed antimicrobial activity and ability to suppress aflatoxins production (Ferreira et al., 2013).

Out of 110 species of genus *Curcuma*, only ∼20 species were used so far for phytochemical studies (Nahar and Sarker, 2007). *Curcuma longa* L. is popularly known as turmeric, while *C. aromatica* Salisb. and *C. caesia* Roxb. are known as wild turmeric and black turmeric, respectively. *C. longa* L. and a few other species, including *C. aromatica* Salisb., produce curcumin, a yellow colored curcuminoid. So far, at least 235 compounds, primarily phenolics, terpenoids, and alkaloids, were identified from *Curcuma* spp. (Li et al., 2011). About 70 varieties of *C. longa* L. are cultivated in India (Sasikumar, 2005; Parthasarathy and Chempakam, 2008), but very few are chemically profiled.

The essential oil of *Curcuma* spp. is used in traditional medicine for many ailments (Dosoky and Setzer, 2018). The volatile component of *C. longa*’s rhizome is responsible for its aromatic flavor and odor (Gounder and Lingamallu, 2012). Its essential oil is considered safe for human use (Tisserand and Young, 2013). The oils of *C. longa* L. and *C. aromatica* Salisb. have applications in the food and pharmaceutical industries due to their antioxidant, antibacterial, and anti-inflammatory properties (Dosoky and Setzer, 2018). The essential oil also improved the bioavailability of curcumin, thereby its bioactivity (Shishu and Maheshwari, 2010). Preliminarily clinical trials indicated that the essential oil from *C. longa* L. and *C. aromatica* Salisb. was helpful against cancer, asthma, and other ailments (Cheng et al., 1999; Joshi et al., 2003; Li Y. et al., 2009). Thus, there is a need to identify high-yielding cultivars containing curcuminoids and essential oil.

Since the pharmacological properties of *Curcuma* spp. are dependent on their chemical profiles, studies on the chemical constituents of turmeric/wild turmeric and their essential oils gained significance. Thin-layer chromatography (TLC) is one of the methods employed to quantify curcumin (Setyaningsih et al., 2016) and other curcuminoids (Phattanawasin et al., 2009) in *Curcuma longa* L. A few different techniques used were HPTLC (Pathania et al., 2006; Paramasivam et al., 2009), nuclear magnetic resonance (NMR) spectroscopy (Li W. et al., 2009), and the HPLC method (Kulyal et al., 2016).

Our present study on metabolite profiles would pave the way for metabolomics by providing the identity of several metabolites. Metabolomics is a practical approach for the comprehensive profiling and comparison of metabolites in plant systems (De Vos et al., 2007). It is crucial for quality evaluation and scientific validation of medicinal plants and their products (Mukherjee et al., 2016). Mainly information on secondary metabolites of medicinal plants/spices is of great importance in health, food, and nutrition sectors, due to the antioxidant nature, color, or flavor of these secondary compounds (Beekwilder et al., 2005; Dixon et al., 2006; Hall, 2006). The quality of turmeric and other spices depends on factors, such as cultivation, collection, storage, milling, and processing, apart from genetics and adulteration issues. Therefore, metabolomics provides a practical approach for quality control (Mukherjee et al., 2016; Tetali et al., 2021).

Over the past decade, several methods suitable for large-scale analysis of metabolites in plant extracts were developed (Dixon et al., 2006; Hall, 2006). However, to date, no single analytical method can successfully detect the entire metabolome of higher plants, especially of medicinal and aromatic plants, as they are highly rich in chemically diverse metabolites (Tetali et al., 2021). The GC-MS and LC-MS techniques mutually complement each other in unraveling secondary metabolomes comprising a wide range of volatile and nonvolatile compounds. These compounds belonged to terpenes, phenolic acids, phenylpropanoids, saponins, alkaloids, polyamines, and their derivatives (Huhman and Sumner, 2002; Moco et al., 2006).

Essential oils from different *Curcuma* species, including *C. longa* L. and *C. aromatica* Salisb*,* were studied for their chemical constituents (Choudhury et al., 1996; Angel et al., 2014; Nampoothiri et al., 2015; Dosoky and Setzer, 2018) to establish their variability. Variation in the volatile compositions of *Curcuma* spp. such as *C. longa* L. and *C. zedoaria*, was done using GC-MS (Dosoky et al., 2019). A combination of GC-MS and LC-MS techniques was used for metabolite analysis of *C. domestica* L. (*C. longa* L.) (Herebian et al., 2009). In the present study, the volatile (essential oil) and nonvolatile (total extract) components of the fresh rhizome of the seven cultivars of *Curcuma* spp. were analyzed by the GC-MS and LC-MS techniques. The present study is the first report revealing such detailed metabolite profiles of the selected cultivars to the best of our knowledge. These cultivars, except Alleppey Supreme, are typically cultivated in Telangana and Andhra Pradesh, and these states are among the largest producers of turmeric in India (Parthasarathy and Chempakam, 2008).

Most of the studies worldwide on *Curcuma* spp., for their curative properties, were with *C. longa* L., followed by *C. aromatica* Salisb, *C. aeruginosa* Roxb. (Simoh and Zainal, 2015), and *C. kwangsiensis* S. K. Lee & C. F. Liang (Zeng et al., 2009). Several cultivars exist within these species, which vary in their chemical profiles. The present article is the first attempt to characterize both volatile (essential oil) and nonvolatile (crude extract) components of fresh rhizomes of seven cultivars of *Curcuma* spp. by the GC-MS and LC-MS techniques. Our results using GC-MS and LC-MS analyses revealed high variability in their metabolite profiles of seven cultivars of genus *Curcuma*. We emphasize that such an approach could be exploited to distinguish cultivars for a specific application based on their metabolite profile.

## Materials and Methods

### Materials and Reagents

LC-MS grade methanol, water, and acetonitrile were purchased from Fisher Scientific (Pittsburgh, PA, United States). Ammonium formate, formic acid, 4-fluoro-4′-hydroxy benzophenone (97%), and *n*-hexane were from Sigma-Aldrich, India. Anhydrous sodium sulfate (99.99%) was from Merck Millipore, India.

Fresh rhizomes of four cultivars of *Curcuma longa* L. (Duggirala Red, Prathibha, Salem, and Suguna) and two cultivars of *C. aromatica* Salisb. (Kasturi Araku and Kasturi Avidi) were collected from Turmeric Research Station, Kammarpally, Telangana State, India. Alleppey Supreme cultivar of *C. longa* L. was from the Indian Institute of Spices Research, Marikunnu (IISR) Kozhikode, Kerala, India. The mature rhizome samples were collected during the postharvest season of turmeric (May–Jun) in 2011 and 2012 and cryopreserved at −80°C until extraction and analysis.

### Isolation of Essential Oil by Hydrodistillation for GC-MS Analysis

50 g each of fresh turmeric rhizome of five cultivars of *C. longa* L. cvs. Alleppey Supreme, Duggirala Red, Prathibha, Salem, Suguna, and two cultivars of *C. aromatica* Salisb. cvs. Kasturi Araku and Kasturi Avidi were taken out from a −80°C freezer, made into pieces, and ground in a pestle with a mortar to a fine powder under liquid nitrogen. The powder was subjected to hydrodistillation in a Clevenger-type apparatus for 7 h. The essential oil obtained after distillation was dried over anhydrous sodium sulfate and kept at −80°C until GC-MS analysis.

### GC-MS Running Conditions and Metabolite Identification

The chemical composition of the *Curcuma* spp. essential oil was analyzed by the GC-MS technique using Agilent 7890 A gas chromatograph coupled with a Leco Pegasus HT TOF mass spectrometer equipped with a 29.8 m × 320 µm HP-5MS 5% phenyl methyl siloxane capillary column with 0.25 µm film thickness. The oven temperature was programmed at 65°C for 2 min and then increased from 65 to 90°C at 5°C/min (held for 3 min). Then the temperature was increased from 90 to 103°C (held for 3 min) and from 103 to 150°C (held for 15 min) at 20°C/min and 8°C/min, respectively. The temperature was raised finally from 150 to 280°C at 20°C/min. The injector, interphase, and ion source were maintained at 250°C, 280°C, and 250°C, respectively. The detector voltage was 1500 V. A solvent delay of 2 min was selected. One microliter (diluted with *n*-hexane; 1:10) of essential oil sample was injected into the GC-MS system using split mode (50: 1). Helium was used as a carrier gas at a flow rate of 1 ml/min. GC-MS data were measured at 70 eV; mass scan 40–1000 amu.

The compounds were identified by comparing their mass spectra with the data available in the literature, National Institute of Standards Technology NIST, and Leco-Fiehn Rtx5 libraries. The compounds originated from the GC-MS data file were identified by matching most resembling spectra with the NIST library. Each search produced a hit list of compounds according to match factor or similarity with the library spectra. All the compounds showing similarity more than 70% with the NIST library were selected by the software (Software: Version 4.22 optimized for Pegasus®). Software searches (identifies) compound from their mass spectra and includes MS interpretation programs for analyzing mass spectra based on chemical structure, molecular formula, isotopic pattern, etc. The similarity of 70% or above between the m/z values of the compound detected in the respective cultivar and the MS-libraries’ mass fragmentation pattern was considered as identification. Furthermore, mass spectra of all compounds were also matched with ranges available as per their CAS number. Compounds for which the CAS number was not generated, the PubChem CID was used. Compounds below the similarity level of 70% were not considered and grouped as unknown. The data obtained with the samples collected in 2012 are presented in this article.

### Preparation of Rhizome Extracts for LC-MS Analysis

Samples for LC-MS analysis were prepared by grinding the fresh rhizome to a fine powder in a mortar and pestle under liquid nitrogen. 1 g of the rhizome powder was suspended in 2 ml of MeOH (LC-MS grade). The samples were sonicated for 30 min and centrifuged for 25 min at 1500 rpm, and the supernatants were separated by filtering through a 0.45-µm Nylon filter disk. These extracts were freshly prepared for the analysis. A 200 µl aliquot of the extract was diluted quantitatively with internal standard (IS) 200 µl 4-fluoro-4′- hydroxy benzophenone solution. It was prepared freshly for each analysis by dissolving in methanol for a final concentration of 0.58 mg/ml. The samples were subjected to LC-MS analysis for the complete metabolite profile. The data obtained with the samples collected in 2012 were presented in this article.

### LC-MS/MS Conditions and Metabolite Identification

LC-MS analyses of the crude extract of fresh rhizome of *Curcuma* spp. were performed according to Jiang et al. (2006) using Agilent 6520 Accurate Q-TOF (Agilent Santa Clara, CA), and the column used was Zorbax Eclipse XDB-C 18, 4.6 × 50 mm, 1.8 µ; Mobile phase: A) buffer (5 mM ammonium formate, 0.1% formic acid, in deionized and distilled H_2_O) and B) acetonitrile; gradient (in buffer A): 0–2 min, 5% B; 2–57 min, 5–100% B; 57–60 min, 100% B; 60–65 min, 100–5% B; flow rate: 0.25 ml/min; temperature, 40^o^C; injection volume 5 µl. For the MS detection, Agilent MSD-Trap-SL was equipped with electrospray ionization (ESI) interface as the ion source. The acquisition parameters for the negative mode were: drying N_2_ temperature, 350^o^C, 8 l/min; nebulizer pressure 40 psi; HV capillary 4000 V; skimmer 65.0 V; mass range measured: 110–1700 m/z; Spray voltage: 4 kV; scan rate 1.4. We analyzed the results in both the positive and the negative ion mode acquired by Agilent TOF/Q-TOF mass spectrometry and full MS scan, in the form of total ion current (TIC) chromatogram, and the metabolites were identified based on their MS/MS spectra and fragmentation rules reported previously (Jiang et al., 2006).

## Results

### Essential Oil Content

The oil was obtained by hydro-distillation, in a Clevenger-type apparatus, of the fresh rhizomes of five cultivars (Alleppey Supreme, Duggirala Red, Prathibha, Salem, and Suguna) of *Curcuma longa* L. and two cultivars (Kasturi Araku and Kasturi Avidi) of *C. aromatica* Salisb. The yield of essential oil from the seven cultivars was in the range of 0.74–1.62% on a fresh weight basis, with the highest yield of 1.62% in cv. Kasturi Avidi (*C. aromatica* Salisb.) followed by cv. Alleppey Supreme (*C. longa* L.) with an amount of 1.42% and the lowest yield of 0.74% in cv. Duggirala Red (*C. longa* L.). The essential oil yields from the other five rhizomes were in between these values (Table 1). The oil yields of *C. longa* L. varieties were higher than those of *C. aromatica* Salisb.

**TABLE 1:** Essential oil content and total number of compounds detected by GC-MS in the rhizomes of *Curcuma* species.

Sl. No.	Cultivar (species)	Essential oil (%)	Identified (Reported in *Curcuma* spp. or another plant species)	Unidentified	
				Unknown	Not reported from any plant species
1	Alleppey Supreme	1.42	31	58	111
	(*C. longa* L.)				
2	Duggirala Red	0.74	36	56	108
	(*C. longa* L.)				
3	Prathibha	1.20	44	60	96
	(*C. longa* L.)				
4	Salem	1.00	30	39	131
	(*C. longa* L.)				
5	Suguna	0.80	35	51	114
	(*C. longa* L.)				
6	Kasturi Araku	0.78	29	64	107
	(*C. aromatica* Salisb.)				
7	Kasturi Avidi	1.62	31	60	109
	(*C. aromatica* Salisb.)				

### GC-MS Analysis of Essential Oil

**FIGURE 1:**
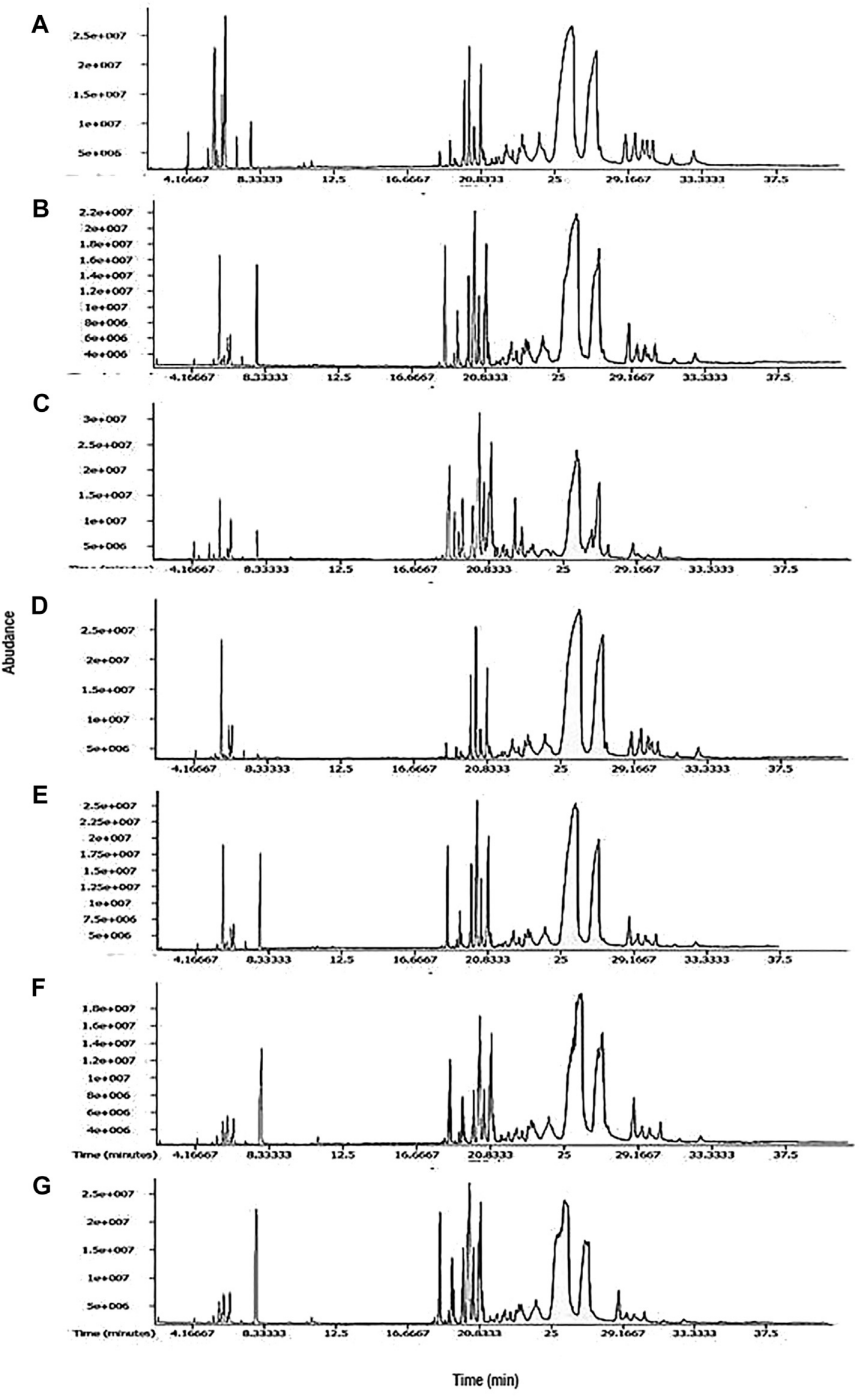
Representative TIC chromatograms from GCMS of essential oil from cultivars **(A)** Alleppey Supreme, **(B)** Duggirala Red, **(C)** Prathibha, **(D)** Salem, **(E)** Suguna of *Curcuma longa* L. and cvs. **(F)** Kasturi Araku, **(G)** Kasturi Avidi of *C. aromatica* Salisb.

Essential oils of seven cultivars of *Curcuma* spp. were subjected to GC-MS analysis, and the results from one of such studies for each cultivar are presented in this article. The representative TIC chromatograms of these cultivars are shown in Figure 1. Several compounds were detected in each cultivar’s essential oil (Table 1). Only a few of the identified compounds were confirmed based on their match with the compound profiles found in the NIST databases and Leco-Fiehn Rtx5 library. Up to 44 compounds were identified from the five cvs. of *C. longa* L. and 31 compounds from two cvs. of *C. aromatica* Salisb. (Table 1). Altogether 80 compounds were grouped into three categories: cultivar-specific (41), present in more than one cultivar (26), and common in all seven cultivars (13).

**FIGURE 2:**
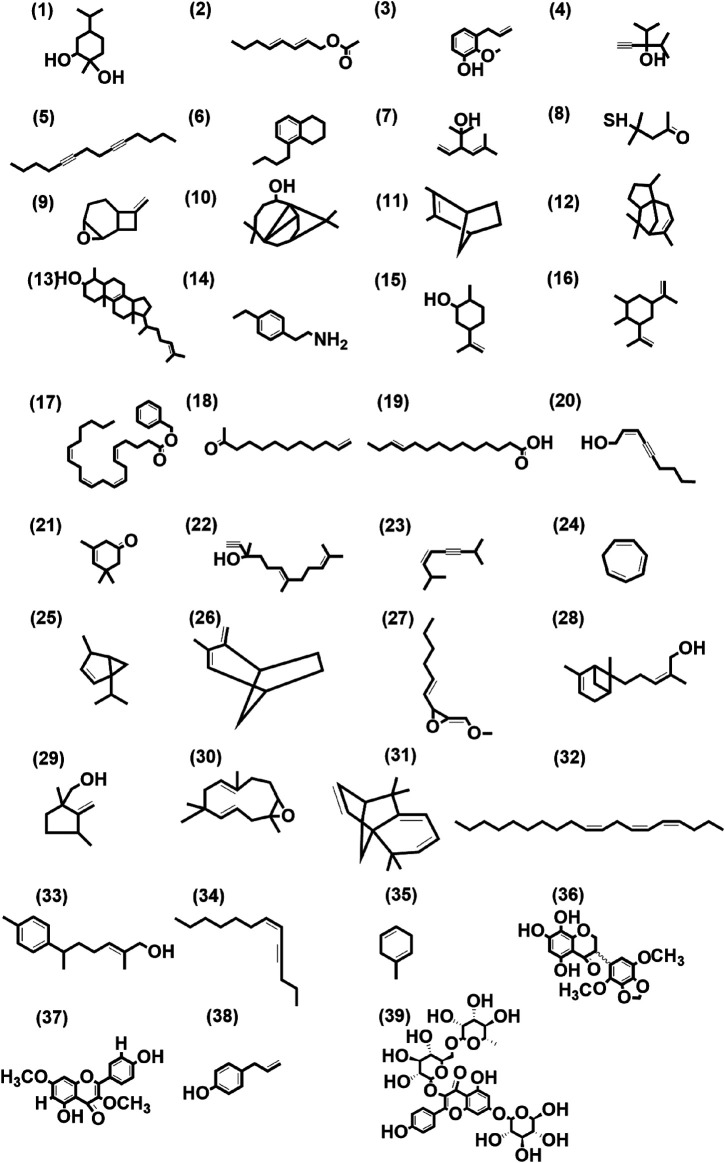
Structures of first-time reported (total 39) from the genus *Curcuma*, identified from cultivars of *Curcuma longa* L. and *C. aromatica* Salisb. detected in essential oil (panel numbers 1–23 and 24–35 corresponding to serial numbers 1–23 and 1–12 of Tables 2, 3 respectively) and rhizome extracts (panel numbers 36 and 37–39 corresponding to serial numbers 1 and 1–3 of Tables 6, 7 respectively) by GC-MS and LC-MS, respectively. Details of all these compounds are given in Supplementary Tables S3, S4. (1) 1,2-Cyclohexanediol, 1-methyl-4-(1-methylethyl)-, (2) trans, trans-octa-2,4-dienyl acetate, (3) phenol, 2-methoxy-3-(2- propenyl)-, (4) 3-isopropyl-4-methyl-1-pentyn-3-ol, (5) 5,9-tetradecadiyne, (6) naphthalene, 5-butyl-1,2,3,4-tetrahydro-, (7) santolina alcohol, (8) 2-pentanone, 4-mercapto-4-methyl-, (9) 8-methylene-3-oxatricyclo[5.2.0.0(2,4)]nonane, (10) 7-tetracyclo[6.2.1.0(3.8)0(3.9)]undecanol, 4,4,11,11 tetramethyl-, (11) bicyclo(2.2.1)hept-2-ene, 2,3-dimethyl-, (12) 1H-3a,7-methanoazulene, 2,3,4,7,8,8a-hexahydro-3,6,8,8-tetramethyl-, [3R-(3à,3aá,7á,8aà)]-, (13) cholesta-8,24-dien-3-ol, 4-methyl-, (3á,4à)-, (14) 4-ethylphenethylamine, (15) cyclohexanol, 2-methyl-5-(1-methylethenyl)-, (16) cyclohexane, 1,2-dimethyl-3,5-bis(1-methylethenyl)-, (17) 5,8,11,14-eicosatetraenoic acid, phenylmethyl ester, (all-Z)-, (18) 11-dodecen-2-one, (19) E-11-tetradecenoic acid, (20) 2-nonen-4-yn-1-ol, (Z)-, (21) 3-cyclohexen-1-one, 3,5,5-trimethyl-, (22) 6,10-dodecadien-1-yn-3-ol, 3,7,11-trimethyl-, (23) 3-octen-5-yne, 2,7-dimethyl-, (Z)-, (24) 1,3,5-cycloheptatriene, (25) bicyclo(3.1.0)hexane, 4-methyl-1-(1-methylethyl)-, didehydro deriv., (26) bicyclo(3.2.1)oct-2-ene, 3-methyl-4-methylene-, (27) oxirane, 2-(hexyn-1-yl)-3-methoxymethylene-, (28) bergamotol, Z-α-trans-, (29) (1,3-dimethyl-2-methylene-cyclopentyl)-methanol, (30) 12-oxabicyclo(9.1.0)dodeca-3,7-diene, 1,5,5,8-tetramethyl-, [1R-(1R*,3E,7E,11R*)]-, (31) isolongifolene, 4,5,9,10-dehydro-, (32) Z,Z,Z-4,6,9-nonadecatriene, (33) 6-(p-tolyl)-2-methyl-2-heptenol, (34) 6-tridecen-4-yne, (Z)-, (35) 1,4-cyclohexadiene, 1-methyl-, (36) kaempferol-3,7-O-dimethyl ether, (37) 5,7,8-trihydroxy-2′,5′-dimethoxy-3′,4′-methylene dioxyisoflavanone, (38) chavicol, (39) kaempferol-3-O-rutinoside-7-O-glucoside.

**FIGURE 3:**
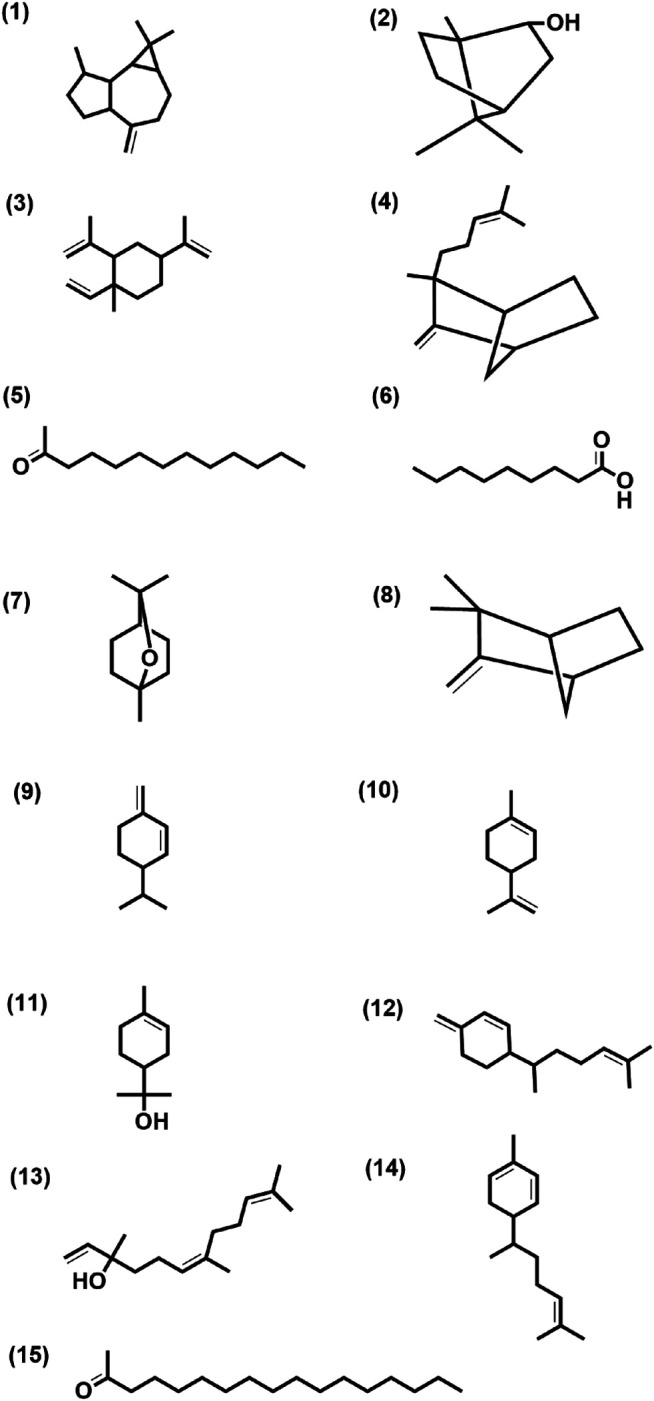
Structure of few selected cultivar-specific (panel numbers 1–7 corresponding to serial numbers 24–30 of Table 2); detected in more than one cultivar (panel numbers 8–13 corresponding to serial numbers 13–18 of Table 3) and common (panel numbers 14–15 corresponding to serial numbers 10 and 13 of Table 4) compounds already reported from the genus *Curcuma* in essential oils isolated from rhizomes by GCMS analysis of seven cultivars of *Curcuma* L.: five of *Curcuma longa* L. (cvs. Alleppey Supreme, Duggirala Red, Prathibha, Salem, and Suguna) and two of *C. aromatica* Salisb. (cvs. Kasturi Araku and Kasturi Avidi). (1) Aromadendrene, (2) isoborneol, (3) β-elemene, (4) α-santalene, (5) 2-tridecanone, (6) nonanoic acid, (7) eucalyptol, (8) camphene, (9) α-phellandrene, (10) limonene, (11) α-terpineol, (12) β-sesquiphellandren, (13) nerolidol, (14) α-zingiberene, (15) 2-heptadecanone.

These 41 cultivar-specific compounds were detected in the essential oil of one of the cultivars of *C. longa* L. or *C. aromatica* Salisb. (Table 2). The essential oil of *C. longa* L. cv. Prathibha had the highest number of cultivar-specific compounds, whereas *C. aromatica* Salisb., cv. Kasturi Araku had the least (Table 2). Of these, 23 cultivar-specific compounds were reported for the first time from the genus *Curcuma*. The chemical structures of these 23 compounds (Table 2, Sl. Nos. 1–23) are presented in Figure 2 (panels 1–23). In addition to 41 cultivar-specific compounds, 26 compounds were present in more than one cultivar (Table 3). Among these, 12 were detected first time in the genus *Curcuma* (Table 3, Sl. Nos. 1 to 12; Figure 2, panels: 24–35). The remaining 14 were already known in *C. longa* L. A total of 13 compounds were common in all seven cultivars of *C. longa* L. and *C. aromatica* Salisb. (Table 4) and the representative structures of two of these compounds are given in Figure 3 (panel numbers 14–15, corresponding to serial numbers 10 and 13 respectively of Table 4). Most of these compounds belong to mono, di, and sesquiterpene. A summary of all 80 compounds identified by GC-MS in the essential oils of the seven cultivars is presented in Supplementary Table S1.

**TABLE 2:** Cultivar-specific compounds identified, in one of the seven cultivars of *Curcuma longa* L. or *C. aromatica* Salisb. by GC-MS in the essential oil from rhizomes. The structures of the compounds (serial numbers from 1 to 23) are given in Figure 2 (panel numbers: 1–23), and this is the first report of these compounds from the genus *Curcuma* L. These compounds, however, were reported from genus other than *Curcuma* L. The compounds from serial numbers 24 to 41 are already reported in *Curcuma* species. Structures for few compounds (serial numbers 24–30) are given in Figure 3 (panel numbers: 1–7). Abbreviations used: AS, Alleppey Supreme; DR, Duggirala Red; PR, Prathibha; SA, Salem; SU, Suguna; KAr, Kasturi Araku; KAv, Kasturi Avidi.

Sl. No.	Compound name	Cultivar	RT (Min)	Area/abundance	Formula	Mass	Mass fragmentations	Class of compound	Reported from plant species	References
1	1,2-Cyclohexanediol, 1-methyl-4-(1-methylethyl)-	AS	6.38	958955880	C_10_H_20_O_2_	172.146	43, 71, 111	Monoterpenoid	*Citrus medica* L.	Bhuiyan et al. (2009)
							154		Leaf and peel essential oil	
2	Trans, trans-Octa-2,4-dienyl acetate	AS	8.22	84981	C_10_H_16_O_2_	168.115	43, 77,79	Dienyl acetate	*Kaempferia galanga* L.	Othman et al. (2006)
									Dried rhizomes	
3	Phenol, 2-methoxy-3-(2-propenyl)-	AS	17.05	200016	C_10_H_12_O_2_	164.083	77,131	Phenolic monoterpenoid	*Dalberga stevensonii* Standl.	Jiang et al. (2018)
							164		Wood extracts	
4	3-Isopropyl-4-methyl-1-pentyn-3-ol	DR	13.59	805101	C_9_H_16_O	140.120	43,97	Alcohol constituent	*Anethum sowa* Roxb. ex*,* Fleming	Saleh-e-in et al. (2010)
							107		Leaf and stem essential oil	
5	5,9-Tetradecadiyne	DR	19.45	11283632	C_14_H_22_	190.172	41,105, 147	Unsaturated hydrocarbon	*Ferula vesceritensis* Coss. & Durieu ex Trab.	Zellagui et al. (2012)
									Leaves	
6	Naphthalene, 5-butyl-1,2,3,4-tetrahydro-	DR	20.62	1290163	C_14_H_20_	188.156	91,145	Tetralin	*Meconopsis punicea* Maxim. and *M. delavayi* (Franch.) Franch. Ex Prain, essential oil	Yuan et al. (2003)
							188	Type		
7	Santolina alcohol	DR	23.63	547909	C_10_H_18_O	154.135	59,81	Tertiary alcohol	*Achillea filipendulina* Lam., aerial part	Sharopov and Setzer (2010)
							121			
8	2-Pentanone, 4-mercapto-4-methyl-	PR	5.32	1330293	C_6_H_12_OS	132.060	43,55	Ketone	*Camellia sinensis* (L.) Kuntze	Kumazawa et al. (2005)
							132			
9	8-Methylene-3-oxatricyclo[5.2.0.0(2,4)]nonane-	PR	11.72	25554	C_9_H_12_O	136.08	40,79, 92	Hydrocarbon	*Schisandra chinensis* (Turcz.) Baill., essential oil dried fruit	Wang et al. (2005)
10	7-Tetracyclo[6.2.1.0(3.8)0(3.9)]undecanol, 4,4,11,11 tetramethyl-	PR	19.16	21297856	C_15_H_24_O	220.182	77,119	Sesquiterpene alcohol	*Cyperus articulatus* L., essential oil roots/rhizome	Metuge et al. (2014)
							159			
11	Bicyclo(2.2.1)hept-2-ene, 2,3-dimethyl-	PR	19.41	23461594	C_9_H_14_	122.109	79,94	Cyclic hydrocarbon	*Abies alba* Mill	Yang et al. (2009)
							122		leaf and twig	
12	1H-3a,7-methanoazulene, 2,3,4,7,8,8a-hexahydro-3,6,8,8-tetramethyl-, [3R-(3à,3aá,7á,8aà)]-	PR	19.69	1483176	C_15_H_24_	204.187	93,119	Sesquiterpene	*Lindera aggregata* (Sims) Kosterm., essential oil	Hong (2011)
							161			
13	Cholesta-8,24-dien-3-ol, 4-methyl-, (3á,4à)-	PR	21.56	84686714	C_28_H_46_O	398.354	69,105	Triterpenoid	*Parkia speciosa* Hassk.	Salman et al. (2006)
							119		seed	
14	4-Ethylphenethylamine	PR	25.88	507899185	C_10_H_15_N	149.120	63,120	Amine	*Psidium guajava* L.	Fasola et al. (2011)
									stem bark essential oil	
15	Cyclohexanol, 2-methyl-5-(1-methylethenyl)-	PR	26.57	3965291	C_10_H_17_O	154.135	67,107	Monoterpenoid	*Mentha spicata* L.	Mohammed et al. (2017)
							136		aerial parts	
16	Cyclohexane, 1,2-dimethyl-3,5-bis(1-methylethenyl)-	PR	26.59	26170712	C_14_H_24_	192.187	107,149	Monoterpenoid	*Rhanterium adpressum* Coss. & Durieu	Kala et al. (2009)
									Aerial parts	
17	5,8,11,14-Eicosatetraenoic acid, phenylmethyl ester, (all-Z)-	SA	5.39	15244294	C_27_H_38_O_2_	394.287	67,91	Mster	*Petiveria alliacea* L., whole plant	Sathiyabalan et al. (2014)
							205			
18	11-Dodecen-2-one	SA	36.83	511982	C1_2_H_22_O	182.167	43,124	Ketone	*Ficus hispida* L. f	Song et al. (2001)
							182		Fresh male and female receptive figs, leaves	
19	E-11-Tetradecenoic acid	SA	37.15	335669	C_14_H_26_O_2_	226.193	41,55,69	Fatty acid	*Coriandrum sativum* L., leaf oil	Bhuiyan et al. (2009)
20	2-Nonen-4-yn-1-ol, (Z)-	SU	10.68	287984	C_9_H_14_O	154.135	41,67	Alcohol	*Alpinia speciosa* (J.C. Wendl.) K. Schum	Ho (2010)
							95, 138		Seeds and leaves	
21	3-Cyclohexen-1-one, 3,5,5-trimethyl-	SU	21.81	10709364	C_9_H_14_O	138.104	96, 138	Cyclohexenone	*Crocus sativus* L.	D’Auria et al. (2006)
									Dried saffron	
22	6,10-Dodecadien-1-yn-3-ol, 3,7,11-trimethyl-	SU	23.27	18320904	C_15_H_24_O	220.182	41,67, 95, 138	Sesquiterpenoid	*Hiptage benghalensis* (L.) Kurz*,* leaves	Venkataramani and Chinnagounder (2012)
23	3-Octen-5-yne, 2,7-dimethyl-, (Z)-	KAv	7.91	132889991	C_10_H_16_	136.125	93, 121, 136	Monoterpene	*Litsea glutinosa* (Lour.) C.B. Rob	Chowdhury et al. (2008b)
									Fruit oil	
24	Aromadendrene	PR	21.68	32839807	C_15_H_24_	204.187	41,67,161	Hydrocarbon	*Curcuma purpurascens* Blume, rhizome, essential oil	Hong et al. (2014)
25	Isoborneol	PR	10.16	931077	C_10_H_18_O	154.135	41,67,95	Monoterpenoid	*Curcuma aromatica* Salisb, rhizome	Sasikumar (2005)
									Essential oil	
26	β-Elemene	PR	17.87	8265459	C_15_H_24_	204.187	41,67193	Sesquiterpene	*Curcuma longa* L., rhizome	Ma and Gang (2006)
									Essential oil	
27	α-Santalene	PR	19.15	167463111	C_15_H_24_	204.187	41,94, 122	Sesquiterpene	*Curcuma longa* L., rhizome	Chowdhury et al. (2008a)
									Essential oil	
28	2-Tridecanone	PR	36.82	145399		198.198	43,57	Ketone	*Curcuma albiflora* Thwaites, rhizome	Herath et al. (2017)
									Essential oil	
29	Nonanoic acid	SU	37.82	1054190	C_9_H_18_O2	158.1307	41,60,129	Fatty acid	*Curcuma longa* L., rhizome	Nieman et al. (2012)
									Essential oil	
30	Eucalyptol	KAr	6.34	73923505	C_10_H_18_O	154.135	51,71, 139	Monoterpene	*Curcuma longa* L., rhizome	Chowdhury et al. (2008a)
									Essential oil	
31	Carvacrol	AS	15.46	800060	C_10_H_14_O	150.104	91, 135	Monoterpene	*Curcuma longa* L., rhizome, essential oil	Awasthi and Dixit (2009)
32	endo-Borneol	PR	25.95	762477	C_10_H_18_O	154.135	95, 140	Monoterpene	*Curcuma longa* L., rhizome	Chowdhury et al. (2008a)
									Essential oil	
33	1,3,5-Cycloheptatriene, 3,7,7-trimethyl-	KAv	22.26	44959671	C_10_H_14_	134.109.120	41,91,119	Cyclic hydrocarbon	*Curcuma longa* L., rhizome	Chowdhury et al. (2008a)
									Essential oil	
34	p-Cymen-8-ol	KAr	11.08	28783214	C_10_H_14_O	150.104	51,91, 135	Monoterpenoid	*Curcuma longa* L., rhizome	Chowdhury et al. (2008a)
									Essential oil	
35	Camphor	PR	9.68	7985363	C_10_H_16_O	152.120	41,95,152	Terpenoid ketone	*Curcuma longa* L., rhizome	Leela et al. (2002)
									Essential oil	
36	α-Bisabolol	PR	22.60	26108003	C_15_H_26_O	222.198	41,69, 119	Sesquiterpenoid	*Curcuma longa* L., rhizome	Chowdhury et al. (2008a)
									Essential oil	
37	α-Elemenone	PR	22.99	5795701	C_15_H_22_O	218.167	67,119, 216	Sesquiterpene	*Curcuma longa* L., rhizome	Singh et al. (2010)
									Essential oil	
38	Caryophyllene oxide	PR	21.85	84236617	C_15_H_24_O	220.182	21,96,138	Sesquiterpenoid oxide	*Curcuma longa* L., rhizome	Chowdhury et al. (2008a)
									Essential oil	
39	Citral	KAr	10.81	4942325	C_10_H_16_O	152.120	69,119	Monoterpene	*Curcuma longa* L., rhizome	Chowdhury et al. (2008a)
									Essential oil	
40	Neoisolongifolene, 8,9-dehydro-	KAr	20.92	956644398	C_15_H_24_	204.187	44,131, 187	Bicyclic hydrocarbon	*Curcuma longa* L., rhizome	Chowdhury et al. (2008a)
									Essential oil	
41	Sabinene hydrate	KAv	19.81	30005835	C_10_H_18_O	154.135	79,93,121	Monoterpene	*Zingiber Officinale* Roscoe, rhizome essential oil	Koo and Gang (2012)

**TABLE 3:** Compounds detected in more than one cultivar of *C. longa* L. and *C. aromatica* Salisb. identified by GCMS in the essential oil from rhizomes. The structures of the compounds from serial numbers 1–12 are given in Figure 2 (panel numbers: 24–35), and the compounds with Sl.No. 13–18 are shown in Figure 3, with corresponding panel numbers: 8–13 respectively. Abbreviations used: AS, Alleppey Supreme; DR, Duggirala Red; PR, Prathibha; SA, Salem; SU, Suguna; KAr, Kasturi Araku; KAv, Kasturi Avidi.

Sl No.	Compound name	Cultivar	RT (Min)	Area/abundance	Formula	Mass	Mass fragment ions	Class of compound	Reported from plant species	References
1	1,3,5-Cycloheptatriene	AS, PR, SA, SU, KAr, KAv	2.16	6243556	C_7_H_8_	92.0626	65,91	Closed ring organic compound	*Ceropegia woodii* Schltr	Meng et al. (2010)
2	Bicyclo(3.1.0)hexane, 4-methyl-1-(1-methylethyl)-, didehydro deriv	DR, PR, SA, SU	5.70	89775296	C_10_H_16_	136.1252	41, 77, 93	Monoterpene	*Zingiber Officinale* Roscoe	Tang et al. (2012)
3	Bicyclo(3.2.1)oct-2-ene, 3-methyl-4-methylene-	DR, SU, KAv	9.35	559996	C_10_H_16_	134.1096	91, 105, 134	Monoterpene	*Seseli daucifolium* C.B. Clarke	Mohiuddin et al. (2012)
4	Oxirane, 2-(hexyn-1-yl)-3-methoxymethylene-	DR, KAr, KAv	9.75	71555	C_10_H_14_O_2_	166.0994	79, 110	Cyclic ether and epoxide	*Hyptis spicigera* Lam	Ladan et al. (2011)
5	Bergamotol, Z-α-trans-	AS, SA	20.90	39326774	C_15_H_24_O	220.182	91, 93,119,187	Sesquiterpene alcohol	*Pogostemon deccanensis* (Panigrahi) Press	Kumar et al. (2019)
6	(1,3-Dimethyl-2-methylene-cyclopentyl)-methanol	AS, DR, SA, SU, KAv	21.05	25037989	C_9_H_16_O	140.1201	67, 77, 94, 109	alcohol H	*Elsholtzia argyi* H. Lév	Peng and Yang (2005)
7	12-Oxabicyclo[9.1.0]dodeca-3,7-diene, 1,5,5,8-tetramethyl-, [1R-(1R*,3E,7E,11R*)]-	DR, KAr	21.67	21304483	C_15_H_24_O	220.1827	67, 96, 109, 138	Epoxide	*Eugenia Caryophyllus* (Spreng.) Bullock & S.G. Harrison	Mani and Boominathan (2011)
8	Isolongifolene, 4,5,9,10-dehydro-	AS, DR, SA, SU, KAr	22.10	350003	C_15_H_20_	200.1565	77, 91, 143, 157, 185	Polycyclic hydrocarbon	*Cymbopogon citratus* (DC.) Stapf	Tajidin (2012)
9	Z,Z,Z-4,6,9-Nonadecatriene	DR, KAv	22.33	169215573	C_34_H_19_	262.2661	79, 93	Hydrocarbon	*Papaver somniferum* L.	Kumaravel et al. (2019)
10	6-(p-Tolyl)-2-methyl-2-heptenol	AS, SU, KAv	22.98	60406564	C_15_H_22_O	218.167	91, 119, 202	Aromatic alcohol	*Zingiber officinale* Roscoe	Choudhari and Kareppa (2013)
11	6-Tridecen-4-yne, (Z)-	DR, PR, SU	23.14	153999724	C_13_H_22_	178.1722	43, 79, 94	Hydrocarbon	*Ambrosia trifida* L.	Wang et al. (2005)
12	1,4-Cyclohexadiene, 1-methyl-	KAr, KAv	23.15	156905042	C_7_H_10_	94.0783	55, 79,94	Aromatic alcohol	*Capsicum annuum* L.	Eggink et al. (2012)
13	Camphene	AS, DR, PR, SU	4.56	930505	C_10_H_16_	136.125	93, 121	Monoterpene	*Curcuma longa* L.	Chowdhury et al. (2008a)
14	α-Phellandrene	AS, DR, SA, SU, KAr	5.72	2645357306	C_10_H_16_	136.125	77, 93	Monoterpene	*Curcuma longa* L.	Chowdhury et al. (2008a)
15	Limonene	AS, SU, KAr, KAv	6.27	202198637	C_10_H_16_	136.125	68, 93	Monoterpene	*Curcuma longa* L.	Singh et al. (2010)
16	α-Terpineol	AS, PR, SA, SU, KAr, KAv	11.25	35019844	C_10_H_18_O	154.135	59	Monoterpenoid	*Curcuma longa* L.	Gopalan et al. (2000)
							93, 121			
17	β-Sesquiphellandren	DR, SA, SU, KAv	20.83	962609070	C_15_H_24_	204.187	68, 79	Sesquiterpene	*Curcuma longa* L.	Chowdhury et al. (2008a)
18	Nerolidol	DR, PR, SA, SU, KAv	21.79	15626126	C_15_H_26_O	222.198	69, 93	Sesquiterpinol	*Curcuma longa* L.	Awasthi and Dixit (2009)
19	Bicyclo(4.1.0)hept-2-ene, 3,7,7-trimethyl-	DR, KAv	5.63	227551	C_10_H_16_	136.125	93, 121	Monoterpene	*Curcuma longa* L.	Chowdhury et al. (2008a)
20	α-Terpinene	AS, DR, SA, KAr, KAv	6.00	28366949	C_10_H_16_	136.125	93, 121, 136	Monoterpene	*Curcuma longa* L.	Chowdhury et al. (2008a)
21	cis-Ocimene	DR, PR, SA, SU, KAr, KAv	6.74	466438	C_10_H_16_	136.125	41, 93	Monoterpene	*Curcuma longa* L.	Usman et al. (2009)
22	γ-Terpinene	AS, DR, PR, SA, SU, KAr	7.01	26650014	C_10_H_16_	136.125	93, 119,136	Monoterpene	*Curcuma longa* L.	*Curcuma longa* L.
										Usman et al. (2009)
23	Linalool	AS, DR, PR, SU	8.15	500594	C_10_H_18_O	154.135	71, 93, 121	Alcohol	*Curcuma longa* L.	*Curcuma longa* L.
										Leela et al. (2002)
24	Terpinene-4-ol	AS, DR, PR, SA, SU	10.83	2557284	C_10_H_18_O	154.135	73, 94, 154	Monoterpene	*Curcuma longa* L.	*Curcuma longa* L.
										Singh et al. (2010)
25	cis-α-Bisabolene	PR, KAr	20.40	11244271	C_15_H_24_	204.187	67, 93, 161, 204	Sesquiterpene	*Curcuma longa* L.	*Curcuma longa* L.
										Chowdhury et al. (2008a)
26	Ar-Tumerone	DR, SA, KAv	25.87	328137262	C_15_H_20_O	216.151	83, 119, 173, 216	Sesquiterpene	*Curcuma longa* L.	*Curcuma longa* L.
										Chowdhury et al. (2008a)

**TABLE 4:** Compounds common in the seven cultivars of *Curcuma* spp detected by GC-MS in essential oil obtained from rhizomes. The structures of the two compounds with serial numbers 10 and 13 are given in Figure 3 (panel numbers: 14–15 respectively).

Sl No.	Compound name	RT (Min)	Area/abundance	Formula	Mass	Mass fragment ions	Class of compound	Reported from plant species	References
1	α-Thujene	4.13	9407392	C_10_H_16_	136.125	93, 136	Monoterpenoid	*Curcuma longa* L.	Raina et al. (2005)
2	1s-α-Pinene	4.27	122103688	C_10_H_16_	136.125	39, 41, 93	Monoterpenoid	*Curcuma longa* L.	Singh et al. (2002)
3	Sabinene	5.05	3901899	C_10_H_16_	136.125	93, 136	Monoterpenoid	*Curcuma longa* L., leaves	Behura et al. (2002)
4	β or m-Cymene	6.20	338719033	C_10_H_14_	134.109	65, 91, 119	Aromatic hydrocarbon	*Curcuma longa* L.	Singh et al. (2002)
5	Terpinolene	7.82	225186296	C_10_H_16_	136.125	93, 121	Monoterpenoid	*Curcuma longa* L.	Leela et al. (2002)
6	trans-α-Bergamotene	18.86	2365810	C_15_H_24_	204.187	69, 93, 119, 161	Sesquiterpene	*Curcuma longa* L.	Raina et al. (2005)
7	α-Caryophyllene	19.23	6097261	C_15_H_24_	204.187	93, 121	Sesquiterpene	*Curcuma longa* L., leaves	Behura et al. (2002)
8	trans-β-Farnesene	19.37	211225438	C_15_H_24_	204.187	69, 93, 133	Sesquiterpene	*Curcuma longa* L.	Singh et al. (2002)
9	Ar-Curcumene	19.88	482956678	C_15_H_22_	202.172	132, 202	Sesquiterpene	*Curcuma longa* L.	Singh et al. (2002)
10	α-Zingiberene	20.25	1117738917	C_15_H_24_	204.187	69, 93, 119, 204	Sesquiterpene	*Curcuma longa* L.	Chowdhury et al. (2008a)
11	Tumerone	22.16	67277186	C_15_H_22_O	218.167	83, 157	Sesquiterpene	*Curcuma longa* L.	Singh et al., 2002)
12	Curlone	27.34	439832546	C_15_H_22_O	218.167	83, 120, 218	Sesquiterpene	*Curcuma longa* L.	Leela et al. (2002)
13	2-Heptadecanone	39.17	152911	C_17_H_34_O	254.261	43, 55, 71, 125	Ketone	*Curcuma angustifolia* ** **Roxb	Srivastava et al. (2006)

### LC-MS Analysis of Methanol Extracts

Methanolic extracts of rhizomes from the seven cultivars of *Curcuma* spp. were subjected to LC-MS analysis. The results from one of such analyses for each cultivar are presented in this article. The use of “positive” and “negative” modes of LC-MS was quite helpful. TIC chromatograms of all seven cultivars of *Curcuma longa* L. and *C. aromatica* Salisb. were shown in Supplementary Figure S1A for negative mode and Supplementary Figure S1B for positive mode.

**FIGURE 4:**
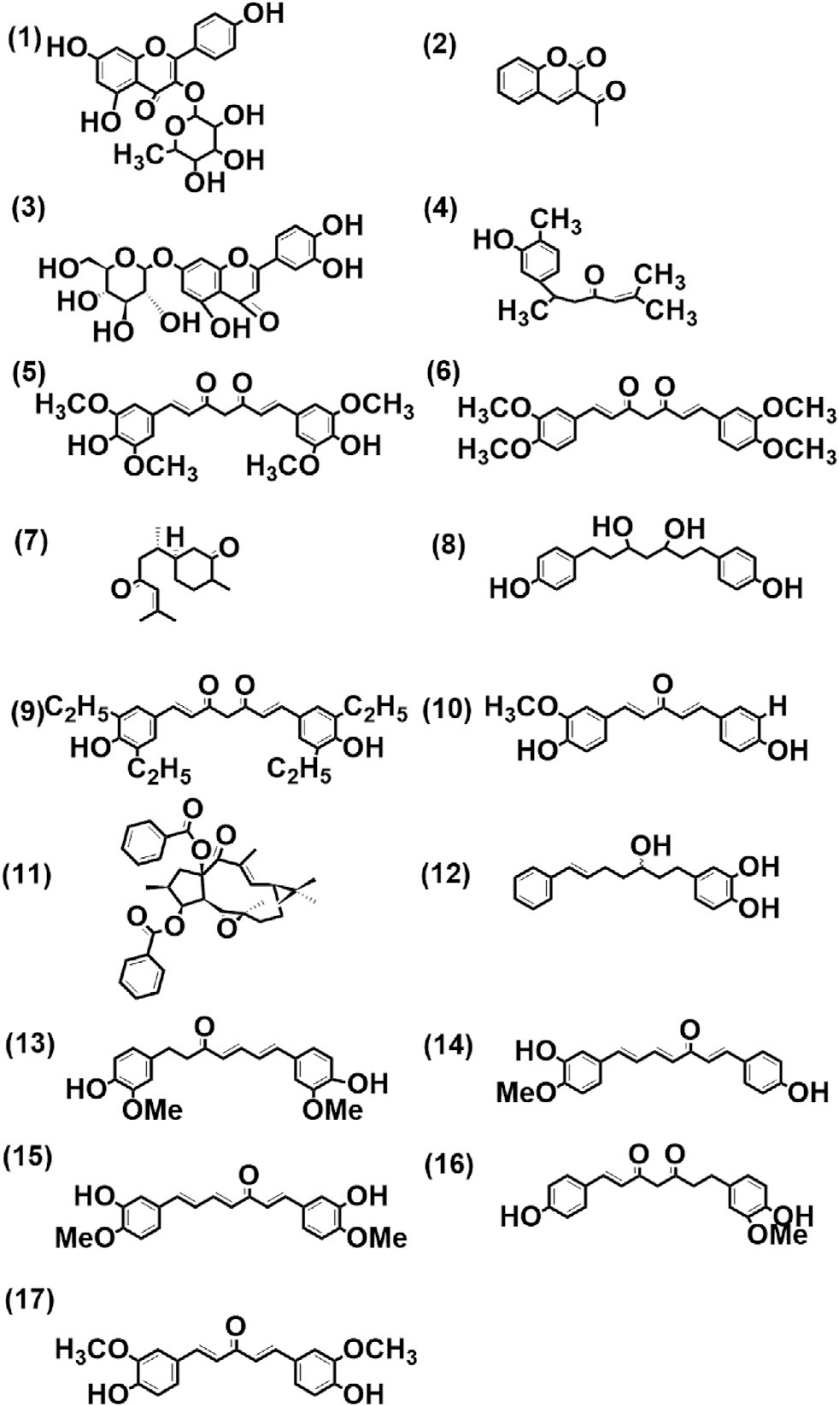
Structure of few selected cultivar-specific (panels 3, 5–11 corresponding to serial numbers 2, 3–9 of Table 6); detected in more than one cultivar (panels 1–2, 4, 12 corresponding to serial numbers 4–5, 6, 7 of Table 7) and common (panels 13, 14–15, 16, and 17 corresponding to serial numbers 10, 5–6, 15, and 1, respectively, of Table 8) compounds already reported from genus *Curcuma* in methanolic extract from rhizomes by LCMS analysis of seven cultivars of *Curcuma* spp.: five of *Curcuma longa* L. (cvs. Alleppey Supreme, Duggirala Red, Prathibha, Salem, and Suguna) and two of *C. aromatica* Salisb. (cvs. Kasturi Araku and Kasturi Avidi). (1) Kaempferol-3-rhamnoside, (2) 3-acetyl coumarin, (3) luteolin-7-O-glucoside, (4) turmeronol, (5) 1,7-bis(4-hydroxy-3,5-dimethoxyphenyl)-1,6-heptadiene-3,5-dione, (6) 1,7-bis(3,4-dimethoxyphenyl)-1,6-heptadiene-3,5-dione, (7) (6S)-2-methyl-6-[(1R,5S)-(4-methene-5-hydroxyl-2-cyclohexen)-2-hepten-4-one, (8) 1,7-bis(4-hydroxyphenyl)-3,5-heptanediol, (9) 1,7-bis(3,5-diethyl-4-hydroxyphenyl)-1,6-heptadiene-3,5-dione, (10) 1-(4-hydroxy-3-methoxyphenyl)-5-(4-hydroxyphenyl)-1,4-pentadiene-3-one, (11) (-)-(12E,2S,3S,4R, 5R,6R, 9S,11S, 15R)-3,15-dibenzoyloxy-5,6-epoxylathyr-12-en-14-one, (12) 7-(3,4-dihydroxyphenyl)-5-hydroxy-1-phenyl-(1E)-1-heptene, (13) 1-(3,4-dihydroxyphenyl)-7-(4-hydroxy-3-methoxyphenyl)-hepta-1,6-diene-3,5-dione, (14) 1-(4-hydroxyphenyl)-7-(4-hydroxy-3-methoxyphenyl)-1,4,6-heptatrien-3-one, (15) 1,7-bis(4-hydroxy-3-methoxyphenyl)-1,4,6-heptatrien-3-one, (16) 1-heptene-3,5-dione, 1,7-bis-(4-hydroxy-3-methoxyphenyl)-, (17) 1,5-bis(4-hydroxy-3-methoxyphenyl)-1,4-pentadien-3-one.

A typical LC-MS analysis of methanolic extracts from rhizomes of *C. longa* L. cv. Alleppey Supreme revealed the presence of up to 86 compounds. Out of these, 43 were identified, and the remaining 43 compounds remained unknown. The (-) ESI-LC-MS detected 30 known compounds, and the (+) ESI-LC-MS detected 23 known compounds with an overlap of 10 compounds, detected by both negative and positive ion modes. A similar assessment of data was done with all seven cultivars of *Curcuma* spp. (Table 5). Altogether 62 compounds were identified, as presented in Supplementary Table S2. These compounds were grouped into three categories: cultivar-specific, detected in more than one cultivar, and common. There were 23 cultivar-specific compounds present in any one cultivar of *C. longa* L. or *C. aromatica* Salisb. (Table 6). 24 compounds were present in more than one cultivar of *C. longa* L. and/or *C. aromatica* Salisb. (Table 7). The remaining 15 were common in all seven cultivars (Table 8). Of these 15 common compounds found in the LC-MS/MS chromatograms, only one was a “Bisabolane” sesquiterpene (Parthasarathy et al., 2009) and all other 14 were diarylheptanoids. These were identified based on the MS/MS spectra reported by Jiang et al. (2006), including curcumin (CU), demethoxycurcumin (DMC), and bisdemethoxycurcumin (BDMC). Among the other diarylheptanoids, 1-(4-hydroxyphenyl)-7-(4-hydroxy-3-methoxyphenyl)-1,4,6-heptatrien-3-one; 1,5-bis(4-hydroxy-3-methoxyphenyl)-1,4-pentadien-3-one, etc., were common in all the cultivars of *C. longa* L. and *C. aromatica* Salisb. (Table 8). The structure of the five common compounds was given in Figure 4 (panels 13, 14–15, 16, and 17 corresponding to serial numbers 10, 5–6, 15, and 1, respectively, of Table 8). In addition to diarylheptanoids, several other classes (phenolic acids, flavonoids, ketonic sesquiterpenes, and fatty acid derivatives) were also detected in the turmeric rhizomes.

**TABLE 5:** Total number of compounds detected by LC-MS from the rhizome extract of *C. longa* L. *and C. aromatica* Salisb.

Sl. No.	Cultivar (Species)	Total number of Metabolites detected	Metabolites identified	Unknown Metabolites
1	Alleppey Supreme	86	43	43
	(*C. longa* L.)			
2	Duggirala Red	107	23	84
	(*C. longa* L.)			
3	Prathibha	60	28	32
	(*C. longa* L.)			
4	Salem	91	28	63
	(*C. longa* L.)			
5	Suguna	96	30	66
	(*C. longa* L.)			
6	Kasturi Avidi	90	30	60
	(*C. aromatica* Salisb.)			
7	Kasturi Araku	92	29	63
	(*C. aromatica* Salisb.)			

**TABLE 6:** Cultivar-specific compounds identified by LC-MS in the rhizome extracts from one of the seven cultivars of *Curcuma longa* L. and *C. aromatica* Salisb. The structure of the compound with the serial number “1” is given in Figure 2 (panel number: 36) and compounds with Sl. Nos. 2, 3–9 are given in Figure 4 with the corresponding panel nos. 3, 5–11, respectively. Abbreviations used: AS, Alleppey Supreme; DR, Duggirala Red; PR, Prathibha; SA, Salem; SU, Suguna; KAr, Kasturi Araku; KAv, Kasturi Avidi.

Sl. No.	Compound name	Cultivar	RT (Min)	Area/abundance	Formula	Mass (m/z)	Mass fragment ions	Class of compound	Reported from plant species	References
1	Kaempferol-3,7-O-dimethyl ether	AS	12.77	25537	C_17_H_14_O_6_	313.0721 M-H^-^	108; 123; 152; 153	Flavonoid	*Lumnitzera racemosa* Willd and	Nikolova (2006); DeSouza et al. (2010)
							167		*Artemisia vulgaris* L.	
2	Luteolin-7-O-glucoside	AS	42.2	5324	C_21_H_20_O_11_	447.2733	110; 185; 279; 280	Flavonoid	*Curcuma Zedoaria* (Christm.) Roscoe	Mass bank ACCESSION:TY00-0145; Rahmatullah et al. (2012)
						M-H				
3	1,7-Bis(4-hydroxy-3,5-dimethoxyphenyl)-1,6-heptadiene-3,5-dione	PR	32.9	186331	C_23_H_24_O_8_	428.2	109; 123; 137; 159; 191; 209	Diarylheptanoid	Curcuma longa L.	Nurfina et al. (1997)
						M-H				
4	1,7-Bis(3,4-dimethoxy phenyl)-1,6-heptadiene-3,5-dione	PR	38.3	335360	C_23_H_24_O_6_	396.2	105; 107; 119; 129; 137; 145; 155; 195	Diarylheptanoid	*Curcuma longa* L.	Chen et al. (2006)
						M-H				
5	(6S)-2-Methyl-6-[(1R,5S)-(4-methene-5-hydroxyl-2-cyclohexen)-2-hepten-4-one	PR	40.2	229164	C_15_H_24_O_2_	236.1	105; 106; 107; 108; 109; 119; 120; 121; 133; 135	Bisabolane	*Curcuma longa* L.	Li et al. (2011)
						M-H				
6	1,7-Bis(4-hydroxyphenyl)-3,5-heptanediol	KAr	22.8	8120	C_19_H_24_O_4_	316.1	105; 106; 107; 119; 121; 147; 148	Diarylheptanoid	*Curcuma longa* L.	Ma and Gang (2006)
						M-H				
7	1,7-Bis(3,5-diethyl-4-hydroxyphenyl)-1,6-heptadiene-3,5-dione	KAr	60.6	129145	C_27_H_32_O_4_	420.3	106; 107; 119; 120; 121; 122; 123; 144; 145; 146; 147; 171; 172; 175; 187; 197; 201; 209; 211; 213; 223; 237; 239; 321; 323	Aromatic	*Curcuma longa* L.	Li et al. (2011)
						M-H				
8	1-(4-Hydroxy-3-methoxyphenyl)-5-(4-hydroxyphenyl)-1,4-pentadiene-3-one	KAv	28.8	8009	C_18_H_16_O_4_	296.1	105; 109; 117; 119; 133; 145; 159; 161; 171; 173; 181; 185; 1207; 209; 223; 233; 239; 251; 279	Diarylheptanoid	*Curcuma longa* L.	Park and Kim (2002)
						M-H				
9	(-)-(12E,2S,3S,4R, 5R,6R, 9S,11S, 15R)-3,15-Dibenzoyloxy-5,6-epoxylathyr-12-en-14-one	KAv	39.8	5683	C_34_H_38_O_6_	542.2	119; 145; 183; 211; 212; 237	Diterpenoid	*Euphorbia micractina* Boiss	Tian et al. (2011)
						M-H				
10	5’-methoxycurcumin	PR	35.56	388655	C_22_H_22_O_7_	398.1	117; 119; 129; 137; 145; 149; 161; 175; 207	Diarylheptanoid	*Curcuma longa* L.	Ravindran (2000)
						M-H				
11	Methyl-7-methoxycoumarin,4-	SU	34.4	67927	C_11_H_10_O_3_	190.1953	115; 116; 117; 119; 120	Coumarin	none	NIST CAS register No. 2555–28–4
										Mass Bank
										ACCESSION: PR100013
12	Hydroferulic acid	KAr	16.8	13992	C_10_H_12_O_6_	195.10	109; 121; 122	Phenolic acid	*Curcuma longa* L.	Ma and Gang (2006)
						M-H				
13	1,2,3,4-Tetraphenylbutane-2,3-diol	KAr	11.1	3926	C_28_H_26_O_2_	394.1	112; 129; 133; 180; 207; 243; 247; 263; 339	Aliphatic diol	none	Pubchem Compound ID: 344369
						M-H				
14	4-Hepten-3-one, 5-hydroxy-1,7-bis(4-hydroxyphenyl)-	PR	25.2	1418	C_19_H_20_O_4_	312.1	118; 119; 120; 146; 161	Diarylheptanoid	*Curcuma longa* L.	Jiang et al. (2006)
						M-H				
15	5,7-Dihydroxy-2-(4-hydroxyphenyl)-chroman-4-one	PR	26.8	12833	C_15_H_12_O_5_	272.0	107; 119; 120	Phenolic acids	none	Chromadex
						M-H				
16	Tetradecanoic acid/myristic acid	AS	29.47	2381	C_14_H_28_O_2_	228.0	128; 130; 143; 155; 158; 182; 183; 184; 210	Fatty acid	none	NIST CAS
						M-H				544–63–8
17	1-(4-Hydroxy-3-methoxyphenyl)-7-(4-hydroxy-3,5-dimethoxypheny)-4,6-heptadiene-3-one	PR	32.0	194388	C_22_H_24_O_6_	384.1	150; 151; 158; 165	Diarylheptanoid	*Curcuma longa* L.	Jiang et al. (2006)
						M-H and M + H				
18	Tumerone	AS	35.3	6120	C_15_H_22_O	218.0	101; 103; 117; 129; 130; 131; 132; 133; 134; 145; 157; 146; 157; 158; 173; 201	Bisabolane sesquiterpene	*Curcuma longa* L.	He (2000)
						M-H				
19	4-Methylene-5-hydroxybisabola-2,10-diene-9-one	PR	44.6	321246	C_15_H_22_O_2_	234.1	105; 107; 115; 117; 119; 121; 122; 129; 135	Sesquiterpene	*Curcuma heyneana* Valeton & Zijp	Sirat and Meng (2009)
						M + H				
20	1,7-Bis(3,4-dimethoxyphenyl)-4,4-dimethyl-1,6-heptadiene-3,5-dione	PR	54.4	9854	C_25_H_28_O_6_	447.2	286; 298; 316; 317	Diarylheptanoid	*Curcuma longa* L.	Liang et al. (2009)
						M-H				
21	25-Benzylpentacyclo-(22.3.1.0.)-octacosa-1(27),3 (8),4,6,10(15),11,13,17(22), 18,20, 24(28), 25-dodecaen	AS	54.96	42646	C_35_H_30_	450.2	105; 107; 119; 121; 122	Cyclic diarylheptanoid	*Myrica rubra* (Lour.) Siebold & Zucc	Ishida et al. (2002)
						M + H				
22	Palmitic acid	SA	59.5	16809	C_16_H_32_O_2_	256.2	120; 166	Fatty acid	*Curcuma kwangsiensis* S.K. Lee & C.F. Liang	Jiang et al. (1989)
						M-H				
23	Stearic acid	SA	63.8	76620	C_18_H_36_O_2_	284.1	197; 199; 200	Fatty acid	*Curcuma longa* L.	Ma and Gang (2006)
						M-H				

**TABLE 7:** Compounds identified by LC-MS in rhizome extracts of more than one cultivar of *Curcuma longa* L. and *C. aromatica* Salisb. The compounds from serial numbers 1–3 are reported first time from the genus *Curcuma*, the structures of these compounds along with few others are given in Figure 2 (panels: 37–39; panels 1–2, 4, 12 corresponding to serial numbers 4–5, 6, 7). Abbreviations used: AS, Alleppey Supreme; DR, Duggirala Red; PR, Prathibha; SA, Salem; SU, Suguna; KAr, Kasturi Araku; KAv, Kasturi Avidi.

Sl. No.	Compound name	Cultivar	RT (Min)	Area/abundance	Formula	Mass (m/z)	Mass fragment ions	Class of compound	Reported from plant species	References
1	5,7,8-Trihydroxy-2′,5′-dimethoxy-3′,4′-methylene dioxyisoflavanone	AS, DR, KAv	2.2	51978	C_18_H_18_O_9_	377.1059	101; 102; 113; 119; 161; 163; 228; 336	Flavonoid	*Terminalia ivorensis* A. Chev	Ogundare and Olajuyigbe (2012)
						M-H^-^				
2	Chavicol	AS, SU	38.83	55821	C_9_H_20_O	135.0794 M + H^+^	102; 115	Terpenoid	*Piper betle* L.	NIST CAS No: 501–92–8
							116			Nagori et al. (2011)
3	Kaempferol-3-O-rutinoside-7-O-glucoside	PR, SA	33.2	3752	C_33_H_40_O_20_	755.2655	135; 161; 175; 176; 191; 439; 579; 755; 756	Flavonoid	*Lycopersicon esculentum* Mill	Le Gall et al. (2003)
						M- H^-^				
4	Kaempferol-3-rhamnoside	AS, DR	10.76	5367	C_21_H_20_O_10_	431.9911	125; 142; 146; 150	Flavonoid	*Curcuma Xanthorrhiza* Roxb	Ruslay et al. (2007)
						M-H	176; 184			
							190; 293; 304; 308			
							311; 316; 343; 345			
5	3-Acetyl coumarin	AS, PR, SA, SU, KAv	34.65	289624	C_11_H_8_O_3_	188.0534	115; 116; 117; 118	Coumarin	None	Pub Chem ID
							141; 143			24852845
										NIST 3949–36–8
6	Turmeronol	AS, DR, SU	25.36	87260	C_15_H_20_O_2_	232.1436	103; 104; 105	Bisabolane sesquiterpene	*Curcuma longa* L.	Ma and Gang (2006)
							107; 115; 117; 118			
							119; 120; 121; 128			
							129; 131; 141; 142; 143			
7	7-(3,4-Dihydroxyphenyl)-5-hydroxy-1-phenyl-(1E)-1-heptene	AS, KAv	35.99	16570	C_19_H_22_O_3_	298.1	119; 183; 184	Diarylheptanoid	*Curcuma xanthorrhiza* Roxb	Suksamrarn et al. (1994)
						M-H				
8	1,7-Diphenyl-1,6-heptadiene-3,5-dione	AS, SA	2.49	9697	C_19_H_16_O_2_	276.0	115	Diarylheptanoid	Synthesized the compound	Sundaryono et al. (2003)
9	1-Hepten-3-one, 5-hydroxy-1,7-bis(3,4-dihydroxyphenyl)-	PR, SA, SU, KAr, KAv	21.2	1660	C_19_H_20_O_6_	344.1	107; 121; 134; 135; 136; 159; 161; 162; 177; 178	Diarylheptanoid	*Alnus japonica* (Thunb.) Steud	Sati et al. (2011)
10	4-(*p*-Hydroxyphenyl)-3-buten-2-one	AS, DR, SA, KAv	22.16	18641	C_10_H_10_O_2_	162.0	117; 118	Flavonoid	None	NIST CAS 3160–35–8
11	5-Hydroxy-7-(4-hydroxyphenyl)-1-phenyl-(1E)-1-heptene	AS, DR, PR, SA, KAr, KAv	23.11	6169	C_20_H_24_ O_4_	328.1	107; 119; 133; 134; 135; 136; 159; 161; 162; 177; 178	Diarylheptanoid	*Curcuma xanthorrhiza* Roxb	Suksamrarn et al. (1994)
12	1-(4-Hydroxy-3-methoxyphenyl)-7-(4-hydroxy-3,5-dimethoxypheny)-4,6-heptadiene-3-one	AS, DR, SA, KAr, KAv	25.8	6408	C_22_H_24_O_6_	384.1	133; 134; 147; 148; 150; 151; 158; 165; 175; 176; 186; 187; 188; 189; 203; 204; 232	Diarylheptanoid	*Curcuma longa* L.	Jiang et al. (2006)
13	1,5-Bis(3,4-methylenedioxyphenyl)-1,4-pentadien-3-one	SU, KAv	26.0	9161	C_19_H_14_O_5_	322.0	115; 119; 121; 133; 143; 235; 237; 247; 263; 275	Diarylheptanoid	*Curcuma longa* L.	Jiang et al. (2006)
14	1-Hydroxy-1-(3,4-dihydroxyphenyl)-7-(4-hydroxy-3-methoxyphenyl)-6-hepten-3,5-dione	AS, KAv	26.7	45589	C_20_H_20_O_7_	372.1	103; 117; 131; 137; 143; 145; 149; 163; 177	Diarylheptanoid	*Curcuma longa* L.	Jiang et al. (2006)
15	1,7-Bis(4-hydroxyphenyl)-1-heptene-3,5-dione	AS, DR, PR, SA, SU, KAv	27.4	8495	C_19_H_18_O_4_	310.1	117; 118; 119; 145; 146; 161; 175; 176	Diarylheptanoid	*Curcuma longa* L.	Jiang et al. (2006)
16	1,7-Bis(4-hydroxyphenyl)-1,4,6-heptatrien-3-one	AS, DR, PR, SA, SU, KAv	30.0	7240	C_19_H_16_O_3_	292.1	115; 117; 119; 120; 143; 145	Diarylheptanoid	*Curcuma longa* L.	Li et al. (2011)
17	7-(4-Hydroxy-3-methoxyphenyl)-1-(4-hydroxy phenyl)-4,6-heptadien-3-one	AS, KAv	31.8	138675	C_20_H_20_O_4_	324.1	107; 117; 119; 120; 122; 123; 131; 135; 137; 145; 146; 147; 148; 163; 195; 223	Diarylheptanoid	*Curcuma longa* L.	Jiang et al. (2006)
18	Coumaran	AS, DR, SA, SU	34.36	6563	C_8_H_8_O	120.0	116; 117; 118; 119	Coumarin	None	Chromadex
19	5,7-Dihydroxy-4-methylcoumarin	AS, SA, SU, KAv	34.57	92188	C_10_H_8_O_4_	192.17	113; 115; 116; 117; 118; 141	Coumarin	None	PubChem CID
										5354284
20	1,7-Bis(3,4,5-trimethoxyphenyl)-l,6-heptadiene-3,5-dione	PR, SU, KAr, KAv	35.82	4972	C_25_H_28_O_8_	456.3	280; 281; 298; 299	Diarylheptanoid	Synthesised the compound	Hahm et al. (2002)
21	Curlone	AS, DR, PR, SU, KAr, KAv	46.50	19540	C_15_H_22_O	218.0	101; 103; 117; 129; 130; 131; 132; 133; 134; 145; 157; 146; 157; 158; 173	Bisabolane sesquiterpene	*Curcuma longa* L.	He (2000)
22	Hydrocinnamic acid	SU, KAr	35.1	3812	C_9_H_10_O_2_	150.0	103; 105; 133	Phenolic acid	*Curcuma longa* L.	Ma and Gang (2006)
23	Curcumenol	AS, SU, KAr, KAv	36.67	34556	C_15_H_22_O_2_	234.1	105; 107; 109; 117; 123; 125; 133; 137	Sesquiterpene	*Curcuma heyneana* Valeton & Zijp	Sirat and Meng (2009)
24	Oleic acid	SA, KAr	60.2	15974	C_18_H_34_O_2_	282.1	168; 257	Fatty acid	*Curcuma longa* L.	Ma and Gang (2006)

**TABLE 8:** Compounds commonly detected by LC-MS analysis of rhizomes extract of all seven cultivars of *Curcuma* spp.: five of *Curcuma longa* L. (cvs. Alleppey Supreme, Duggirala Red, Prathibha, Salem, and Suguna) and two of *C. aromatica* Salisb. (cvs. Kasturi Araku, Kasturi Avidi). The structures for the compounds in the serial numbers 10, 5, 6, 15, and 1 are given in Figure 4 (panels 13, 14, 15, 16, and 17 respectively).

Sl. No.	Compound name	RT (Min)	Area/abundance	Formula	Mass (m/z)	Mass fragment ions	Class of compound	Reported from plant species	References
1	1,5-Bis(4-hydroxy-3-methoxyphenyl)-1,4-pentadien-3-one	23.5	3535	C_19_H_18_O_5_	325.1247	117; 118; 119; 120	Diarylheptanoid	*Curcuma longa* L.	Li et al. (2011)
						135; 143; 145; 146			
						159; 161; 187			
2	Ar-Turmerone	24.7	23545	C_15_H_20_O	216.1	103; 104; 105; 106; 107; 108; 115; 116; 117; 118; 119; 120	Bisabolane sesquiterpene	*Curcuma longa* L.	Parthasarathy and Chempakam (2008)
3	Tetrahydroxybisdemethoxycurcumin	25.4	4773	C_19_H_20_O_4_	311.1446	117; 118; 119; 120	Diarylheptanoid	*Curcuma longa* L.	Jiang et al. (2006)
						146; 161			
4	Tetrahydrodemethoxycurcumin	25.9	7734	C_20_H_22_O_5_	341.1272	101; 113; 119	Diarylheptanoid	*Piper nigrum* L.	Ravindran (2000)
5	1-(4-Hydroxyphenyl)-7-(4-hydroxy-3-methoxyphenyl)-1,4,6-heptatrien-3-one	26.1	76458	C_20_H_18_O_4_	321.0938	115; 117; 119; 121	Diarylheptanoid	*Curcuma longa* L.	Jiang et al. (2006)
						132; 133; 134; 143			
						145; 174; 235; 237			
						247; 263’264; 274			
						275			
6	1,7-Bis(4-hydroxy-3-methoxyphenyl)-1,4,6-heptatrien-3-one	26.3	64172	C_21_H_20_O_5_	351.1123	108; 115; 119; 136	Diarylheptanoid	*Curcuma longa* L.	Jiang et al. (2006)
						143; 148; 164; 195			
						207; 223; 224; 235			
						245; 251; 261; 262			
						263; 279; 291; 307			
7	Tetrahydroxycurcumin	26.5	81260	C_20_H_20_O_7_	371.1686	133; 134; 135; 148	Diarylheptanoid	*Curcuma longa* L.	Jiang et al. (2006)
						149; 175; 176; 177			
8	1-(4-Hydroxy-3-methoxyphenyl)-7-(4-hydroxy-3,5-dimethoxyphenyl)-1,4,6-heptatrien-3-one	26.84	58571	C_22_H_22_O_6_	382.1	149; 159; 173; 197; 209; 211; 221; 233; 237; 239; 249; 261; 267; 277; 289; 293; 295; 305; 309	Diarylheptanoid	*Curcuma longa* L.	Jiang et al. (2006)
9	1,6-Heptadiene-3,5-dione, 1-(3,4-dihydroxyphenyl)-7-(4-hydroxy phenyl)-	31.6	5753	C_19_H_16_O_5_	324.1	134; 135; 136; 143	Diarylheptanoid	*Curcuma longa* L.	Jiang et al. (2006)
10	1-(3,4-Dihydroxyphenyl)-7-(4-hydroxy-3-methoxyphenyl)-hepta-1,6-diene-3,5-dione	32.3	16375	C_20_H_18_O_6_	353.1212	134; 135; 136; 150	Diarylheptanoid	*Curcuma longa* L.	Jiang et al. (2006)
11	Bisdemthoxycurcumin	34.5	1675644	C_19_H_16_O_4_	307.1132	117; 119; 120; 143	Diarylheptanoid	*Curcuma longa* L.	Jiang et al. (2006)
						145			
12	Demethoxycurcumin	35.4	1302410	C_20_H_18_O_5_	337.1251	117; 119; 120; 132	Diarylheptanoid	*Curcuma longa* L.	Jiang et al. (2006)
						134; 143; 145; 158			
						160; 175; 201			
13	Dihydrocurcumin	35.8	5151	C_21_H_22_O_6_	369.1533	132; 134; 135; 149	Diarylheptanoid	*Curcuma longa* L.	Jiang et al. (2006)
						158; 160; 175			
14	Curcumin	36.2	1503497	C_21_H_20_O_6_	367.1374	132; 133; 134; 135	Diarylheptanoid	*Curcuma longa* L.	Jiang et al. (2006)
						149; 158; 160; 161			
						175			
15	1-Heptene-3,5-dione, 1,7-bis-(4-hydroxy-3-methoxyphenyl)-	43.2	5078	C_20_H_20_O_5_	339.1473	117; 119; 120; 134	Diarylheptanoid	*Curcuma longa* L.	Jiang et al. (2006)
						158			

### Compounds Reported First Time From the Genus Curcuma Using GC-MS and LC-MS Analysis

A total of 39 compounds were detected (Figure 2) for the first time from the genus *Curcuma*. Out of these, 35 and 4 compounds were identified respectively in the essential oils and whole rhizome extracts of *C. longa* L. and *C. aromatica* Salisb. by the GC-MS and LC-MS techniques. Details of the compounds, including the class of compound, molecular weight, are given in Tables 2, 3, 5, and 6; structures of all these compounds are shown in Figure 2 (panels: 1–39). The MS and MS/MS spectra of these compounds are presented in Supplementary Figure S2 (panels: 1–39). These compounds were reported earlier from plants belonging to any genus other than *Curcuma,* and this is the first report from genus *Curcuma*. Out of the total of 62 compounds detected by LC-MS analyses of rhizome extracts, four compounds were reported for the first time from the *Curcuma* genus. One was cultivar-specific (Table 6; Sl. Nos. 1; Figure 2, panels: 36), and three were present in more than one cultivar (Table 7; Sl. Nos. 1–3). The structures of these first-time reported compounds are given in Figure 2 (panels: 37–39), and their corresponding mass fragmentation spectra are shown in Supplementary Figure S2 (panels: 36–39).

A comprehensive table each for GCMS (Supplementary Table S3) and LCMS (Supplementary Table S4) shows the details of compound identification methods used in the present study for the first-time reported compounds from genus *Curcuma* and the previous literature. A list of 80 (GC-MS) and 62 (LC-MS) compounds can be seen in Supplementary Tables S5, S6 respectively.

## Discussion

In one of our previous studies, we reported that the HPLC method could be a valuable tool to differentiate the cultivars of *Curcuma* spp. based on their curcuminoids content ratios (Kulyal et al., 2016). Curcuminoids play a significant role in food, cosmetics, and medicinal compounds. But there are several other secondary metabolites such as terpenoids (e.g., mono-, sesqui-, di-, tri-, so on), alkenes, aromatic compounds, flavonoids, coumarins, etc. that are responsible for various biological activities. All these secondary metabolites are present in either the volatile essential oil or the nonvolatile fraction of the *Curcuma* spp. Employing untargeted metabolomics would be the ideal way to identify as many metabolites as possible. Therefore, in the present study, we analyzed these secondary compounds using GC-MS and LC-MS/MS.

### Versatility of GC-MS and LC-MS Techniques to Identify a Large Number of Metabolites

GC-MS analysis is an appropriate technique for analyzing volatile compounds, whereas LC-MS is for detecting polar compounds, and thus, these two techniques are mutually complementary to each other. In the present study, several of the volatile compounds present in the cultivars of *C. longa* L. and *C. aromatica* Salisb. belonging to mono- and sesquiterpenoids were detected by GC-MS (Tables 2–4). On the other hand, LC-MS analysis detected phenolic (Tables 6–8) compounds, including several diarylheptanoids in the methanolic extracts of both *C. longa* L. and *C. aromatica* Salisb. (Figure 4). Electrospray ionization (ESI), coupled with LC/MS/MS, turned out to be a powerful tool in metabolite profiling and metabolomics research. Studies on chemical derivatization and quantification of several metabolites in turmeric powders and fresh rhizome extracts by LC-MS or LC-MS/MS were made. But the rapid screening within the cultivars of *C. longa* L. of fresh turmeric rhizome has not yet been reported. To the best of our knowledge, we were able to record the presence of several metabolites, which were not reported so far in the *C. longa* L. and *C. aromatica* Salisb. (Tables 2, 3, 6, 7, and Figure 2), using the available literature search, Metlin library, mass bank, and NIST library.

### Cultivar Variability Based on Secondary Metabolites

Based on the presence or absence of metabolites identified by GC-MS and LC-MS analyses, there was a need to authenticate cultivar variability. Thus, the metabolite library can be constructed based on the cultivar-specific and compounds found in more than one cultivar. There are very few reports on cultivar-specific secondary metabolite variation. Out of a total of 142 compounds identified by both GC-MS and LC-MS, only 28 compounds (13 from GCMS and 15 from LCMS) were common (Tables 4, 8) present in all the cultivars of *C. longa* L. and *C. aromatica* Salisb. Ten of 13 common compounds (GCMS) were reported earlier from *C. longa* L. rhizome. Two compounds, namely sabinene and α-caryophyllene, were reported from the leaves of *C. longa* L. The remaining one compound, i.e., 2-heptadecanone, was detected for the first time from these two Curcuma species. This compound was earlier reported in the essential oil of *Curcuma angustifolia* Roxb. rhizome (Srivastava et al., 2006).

As per our analyses, 64 compounds (Tables 2, 6) out of 142 compounds were cultivar-specific. Of these 64 compounds, 41 were identified in essential oils by GC-MS (e.g., carvacrol, endo-borneol) and 23 (e.g., tumerone, methyl-7-methoxycoumarin,4-) in fresh rhizome extracts (LC-MS) of any one of the cultivars of *C. longa* L. or *C. aromatica* Salisb. In addition, 50 compounds (Tables 3, 7) were identified to be present in some of the cultivars, present in more than one cultivar but not common to all the cultivars of *C. longa* L. and *C. aromatica* Salisb. Out of these 50 compounds, 26 were identified in essential oils through GC-MS. For example, 1,3,5-cycloheptatriene was detected in all six cultivars except cv. Duggirala Red, whereas 12-oxabicyclo(9.1.0)dodeca-3,7-diene, 1,5,5,8-tetramethyl-, [1R-(1R*,3E,7E,11R*)]-, was detected only in cvs. Duggirala Red and Kasturi Araku. The rest 24 compounds were detected in rhizome extracts by LC-MS (e.g., chavicol detected in cvs. Alleppey Supreme and Suguna). The present extensive analyses of both essential oils and whole rhizome secondary metabolome of seven cultivars of *C. longa* L. and *C. aromatica* Salisb. established cultivar variability. Variability of the compounds within or/and in between the cultivars of *C. longa* L. and *C. aromatica* Salisb. will give a better understanding of their selection. The current study will help select cultivars for use in pharmacology or the food industry.

### Discovery of First-Time Reported Metabolites in *C. longa* L. and *C. aromatica* Salisb.

In the present study, as many as 142 compounds were identified in the essential oils and rhizome extracts of *C. longa* L. and *C. aromatica* Salisb. Out of these, 39 compounds were identified for the first time in the genus *Curcuma*. However, these compounds were found in other plant genera. The structures of these compounds are shown in Figure 2, and corresponding details, including the class of compound, molecular weight, are given in Tables 2, 3, 6, and 7. As an example, cv. Alleppey Supreme of *C. longa* L. showed three cultivar-specific compounds. Among these, 1,2-cyclohexanediol, 1-methyl-4-(1-methylethyl)- (oxygenated alcoholic monoterpenoid) was earlier reported from leaf and peel essential oil of *Citrus medica* L. (Rutaceae). This compound is used as a flavoring agent (Bhuiyan et al., 2009); trans, trans-octa-2,4-dienyl acetate, present in common Malaysian *Kaempferia galanga* L. (Zingiberaceae), was used for its food-flavoring property (Othman et al., 2006). Phenol, 2-methoxy-3-(2-propenyl)-, an allyl chain-substituted guaiacol was reported from rosewood extracts (Jiang et al., 2018).

The following four compounds were identified from cv. Duggirala Red (*C. longa* L.): 3-isopropyl-4-methyl-1-pentyn-3-ol (alcohol constituent) containing leaf and stem of *Anethum sowa* Roxb. ex*,* Fleming, used for flavoring of food, beverages and also for many medical preparations (Saleh-e-in et al., 2010); 5,9-tetradecadiyne (unsaturated hydrocarbon) was found to be a major component of *Ferula vesceritensis* Coss. & Durieu ex Trab. leaf essential oil (Zellagui et al., 2012); naphthalene, 5-butyl-1,2,3,4-tetrahydro- (tetralin type of compounds) found in the essential oil of *Meconopsis punicea* Maxim. and *M.delavayi* (Franch.) Franch. Ex Prain (Slavík and Slavíková; Yuan et al., 2003); and santolina alcohol was reported from plant *Achillea filipendulina* Lam. (Sharopov and Setzer, 2010). A total of nine cultivar-specific compounds were detected in cv. Prathibha (*C. longa* L.) and the examples of these compounds and their source plants, respectively, are 7-tetracyclo[6.2.1.0(3.8)0(3.9)]undecanol, 4,4,11,11 tetramethyl- in *Cyperus articulatus* L. (Metuge et al., 2014); bicyclo(2.2.1)hept-2-ene, 2,3-dimethyl- in *Abies alba* Mill. (Yang et al., 2009). Cultivar-specific compounds of three each were detected in cvs. Salem (*C. longa* L.) and Suguna (*C. longa* L.) (Table 2). In *C. aromatica* Salisb., only one cultivar-specific compound was detected in cv. Kasturi Avidi, i.e., 3-octen-5-yne, 2,7-dimethyl-, (Z)-, and this compound was earlier reported from the medicinally important *Litsea glutinosa* (Lour.) C.B. Rob. fruit essential oil (Chowdhury et al., 2008a).

Some of the compounds identified in our study were present in more than one cultivar. For example, 6-(p-tolyl)-2-methyl-2-heptenol (Table 3) was detected in three cvs.: Alleppey supreme, Suguna of *C. longa* L., and Kasturi Avidi of *C. aromatica* Salisb. This compound was earlier reported from *Zingiber officinale* Roscoe (Zingiberaceae), used as a spice, food products, and beverages (Choudhari and Kareppa, 2013).

### Limitations and Strengths of the Present Study

There is significant variability within and between the cultivars of *C. longa* L. and *C. aromatica* Salisb, which can be exploited to differentiate the cultivars of *Curcuma* spp. The feasibility of studies without using any standard compounds was pointed out by Núñez et al. (2020). Similarly, reference compounds were not used in our study to derive arithmetic indices under the experimental conditions. Despite the dilution made in the essential oil sample before injecting into the GC-MS system, the sample was still too concentrated. The high concentration of oil might have restricted the resolution due to overloading the detector. This could be the reason that we could not identify several compounds. We would ensure the further dilution of the oil sample in our future studies. However, the technology employed, GC-TOFMS and LC-QTOFMS, and MS-spectral database/literature search enabled us to establish the cultivar variability of *Curcuma* spp. The detailed information on the metabolite variability within or/and between the cultivars of *C. longa* L. and *C. aromatica* Salisb. may assist us in selecting the cultivars for a specific purpose, like culinary use, coloring, or pharmacological purpose. The studies such as the present one can help to select cultivars, particularly for use in pharmacology or the food industry. Metabolite variability poses a challenge in the use of turmeric in therapy. The practitioners need to be quite careful and use the identified cultivar and avoid mix-up. The caution applies to commercial/industrial use. Once standardized, the protocol should ensure the use of a specific cultivar. Our GC-MS and LC-MS-based metabolite identification is distinct from chemophenetic studies but is a complementary approach to characterize the *Curcuma* metabolome.

### Importance of *Curcuma* spp. Metabolites for Human Health

Curcuminoids (CU, DMC, and BDMC) were identified as the main bioactive compounds of genus *Curcuma* and proved to have a broad spectrum of biological activities based on pharmacological studies. However, rhizomes and their essential oils of *Curcuma* spp. contained several other bioactive (volatile and nonvolatile) compounds. A summary of the pharmacological studies with the metabolites detected in the present study is given in Table 9. Some of the studies demonstrated therapeutic activity with the isolated metabolites, e.g., carvacrol (Suntres et al., 2015), p-cymene (De Oliveira et al., 2015), which are commonly found in essential oils of *Curcuma* spp. A few other reports correlated anti-inflammatory and antioxidant properties of *C. longa* L. essential oil with its chemical components ar-tumerone, α-santalene (Singh et al., 2010) (Table 9). Several compounds detected in the present study in the essential oil or rhizome extracts of *C. longa* L. or *C. aromatica* Salisb. were also found in the essential oil of other medicinal plants, traditionally used for their health benefits. The examples of such compounds are 5,9-tetradecadiyne, a cultivar-specific compound of Duggirala Red (*C. longa* L.), earlier reported in *Ferula vesceritensis* Coss. & Durieu ex Trab. leaf essential oil, exhibiting antibacterial activity; 3-octen-5-yne, 2,7-dimethyl-, (Z)-, a hydrocarbon monoterpene, identified from cv. Kasturi Avidi (*C. aromatica* Salisb.) was earlier reported from fruit essential oil of the medicinally important plant, *Litsea glutinosa* (Lour.) C.B. Rob. (Chowdhury et al., 2008a). We suggest that the medicinal use of the genus *Curcuma* can be not only species but also cultivar-specific.

**TABLE 9:** Pharmacological activity of metabolites identified, other than major curcuminoids (curcumin, demethoxycurcumin, and bisdemethoxy curcumin), in *C. longa* L. and *C. aromatica* Salisb.

Sl. No.	Compound name	Source plant	Tested Compound/essential oil/extract	Pharmacological activity/health benefit of the compound or compound containing plant product	References
					(Activity assay or compound detection)
1	Carvacrol	*Curcuma longa* L.	Compound	Antibacterial	Suntres et al. (2015)
2	p-Cymene	*Curcuma longa* L.	Compound	Antioxidant	(De Oliveira et al. (2015)
				Anti-inflammatory, anticancer, and antimicrobial effects	Marchese et al. (2017)
3	Eucalyptol	*Curcuma longa* L.	Compound	Antitumor anti-inflammatory relevance to Alzheimer’s disease	Murata et al. (2013); Islam et al. (2014)
4	α-Pinene	*Curcuma longa* L.	Compound	Anti-inflammatory and chondroprotective	Rufino et al. (2014)
5	α-Terpineol	*Curcuma longa* L.	Compound	Anti-inflammatory	De Oliveira et al. (2012)
6	Terpinolene	*Curcuma longa* L.	Compound	Anticancer	Okumura et al. (2012)
7	2-Heptadecanone	*Curcuma angustifolia* Roxb	Dried rhizome essential oil	As a coolant, demulscent	Srivastava et al. (2006)
8	Santolina alcohol	*Achillea filipendulina* Lam	Aerial part essential oil	Traditional herbal medicine	Sharopov and Setzer (2010)
9	Cyclohexanol, 2-methyl-5-(1-methylethenyl)-	*Mentha spicata* L.	Aerial parts essential oil	Antifungal	Mohammed et al. (2017)
10	Cyclohexane, 1,2-dimethyl-3,5-bis(1-methylethenyl)-	*Rhanterium adpressum* Coss. & Durieu	Aerial parts essential oil	Antifungal	Kala et al. (2009)
11	4-Ethylphenethylamine	*Psidium guajava* L.	Stem bark essential oil	Antioxidant	Fasola et al., 2011)
12	5,9-Tetradecadiyne	*Ferula vesceritensis* Coss. & Durieu ex Trab	Leaves essential oil	Antibacterial	Zellagui et al. (2012)
13	E-11-Tetradecenoic acid	*Coriandrum sativum* L.	Leaf essential oil	Spice, flavoring agent, antimicrobial	Bhuiyan et al. (2009)
14	6,10-Dodecadien-1-yn-3-ol, 3,7,11-trimethyl-	*Hiptage benghalensis* (L.) Kurz	Leaves essential oil	Treatment of skin diseases, cough, asthma, leprosy	Venkataramani and Chinnagounder (2012)
15	Chavicol	*Piper betle* L.	Leaf oil	Antifungal, antiseptic, and anthelmintic	Nagori et al. (2011)
16	1,2-Cyclohexanediol, 1-methyl-4-(1-methylethyl)-	*Citrus medica* L.	Leaf and peel essential oil	Antibiotic	Bhuiyan et al. (2009)
17	3-Isopropyl-4-methyl-1-pentyn-3-ol	*Anethum sowa* Roxb. ex*,* Fleming	Leaf and stem essential oil	Flavoring of food and beverages, antimicrobial, antioxidant	Saleh-e-in et al. (2010)
18	Bicyclo(2.2.1)hept-2-ene, 2,3-dimethyl-	*Abies alba* Mill	Leaf and twig essential oil	Radical scavenging activity	Yang et al. (2009)
19	2-Nonen-4-yn-1-ol, (Z)-	*Alpinia speciosa* (J.C. Wendl.) K. Schum	Seeds and leaves essential oil	As a food and herbal medicine, mosquito larvicidal activity	Ho (2010)
20	8-Methylene-3-oxatricyclo[5.2.0.0(2,4)]nonane	*Schisandra chinensis* (Turcz.) Baill	Dried fruit essential oil	Antioxidant	Wang et al. (2005)
21	3-Cyclohexen-1-one, 3,5,5-trimethyl-	*Crocus sativus* L.	Dried saffron oil	Antitumor	(D’Auria et al. (200
22	Ar-Tumerone	*Curcuma longa* L.	Rhizome essential oil	Antioxidant	Singh et al. (2010)
	α-Turmerone				
	β-Turmerone				
	α-Santalene				
	Ar-Curcumene				
23	7-Tetracyclo[6.2.1.0(3.8)0(3.9)]undecanol, 4,4,11,11 tetramethyl-	*Cyperus articulatus* L.	Roots/rhizome essential oil	Anti-onchocera activity	Metuge et al. (2014)
24	Cholesta-8,24-dien-3-ol, 4-methyl-, (3á,4à)-	*Parkia speciosa* Hassk.	Seed essential oil	High nutritional and medicinal value	Salman et al. (2006)
25	3-Octen-5-yne, 2,7-dimethyl-, (Z)-	*Litsea glutinosa* (Lour.) C.B. Rob	Fruit essential oil	Antirheumatic	Chowdhury et al. (2008b)
26	5,8,11,14-Eicosatetraenoic acid, phenylmethyl ester, (all-Z)-	*Petiveria alliacea* L.	Whole plant essential oil	Used as folk medicine to enhance memory and in treatment of common cold, flu, other viral, or bacterial infections	Sathiyabalan et al. (2014)
27	Naphthalene, 5-butyl-1,2,3,4-tetrahydro-	*Meconopsis punicea* Maxim. and *M. delavayi* (Franch.) Franch. Ex Prain	Whole plant essential oil	As a traditional medicinal plant for anti-inflammatory and analgesic activity	Yuan et al. (2003)
28	1H-3a,7-methanoazulene, 2,3,4,7,8,8a-hexahydro-3,6,8,8-tetramethyl-, [3R-(3à,3aá,7á,8aà)]-	*Lindera aggregata* (Sims) Kosterm	Root/tubers essential oil	Treatment of decubitus ulcer	Hong (2011)
29	trans, trans-Octa-2,4-dienyl acetate	*Kaempferia galanga* L.	Dried rhizomes	Spice, food-flavoring agent	Othman et al. (2006)
				Vasorelaxant	
30	11-Dodecen-2-one	*Ficus hispida* L. f	Fresh male and female receptive figs	Hepatoprotective, anti-inflammatory, antipyretic	Song et al. (2001)
31	Kaempferol-3,7-O-dimethyl ether	*Lumnitzera racemosa* Willd and *Artemisia vulgaris* L.	Fresh twig methanolic extract; leaf methanolic extract	Antibacterial	Nikolova (2006); DeSouza et al. (2010)
32	5,7,8-Trihydroxy-2′,5′-dimethoxy-3′,4′-methylene dioxyisoflavanone	*Terminalia ivorensis* A. Chev	Fresh sawdust methanolic extract	Antifungal	Ogundare and Olajuyigbe (2012)
33	Kaempferol-3-O-rutinoside-7-O-glucoside	*Lycopersicon esculentum* Mill	Methanolic extract of fruit	Antioxidant	Le Gall et al. (2002)
34	Phenol, 2-methoxy-3-(2- propenyl)-	*Dalberga stevensonii* Standl	Wood extracts	Used as raw material for medical industries	Jiang et al. (2018)
35	2-Pentanone, 4-mercapto-4-methyl-	*Camellia sinensis* (L.) Kuntze	Tea leaves	Antioxidant	Kumazawa et al. (2005)

## Concluding Remarks

Essential oils from spices and aromatic plants are enriched with bioactive metabolites, easily isolated and used, unlike the difficulties encountered with synthetic chemical products. The low mammalian toxicity and biodegradable nature of the natural secondary products provide an attractive option to develop them also for crop protection. Metabolomics is a practical and dynamic approach to make a comprehensive study. Both GC-MS and LC-MS techniques should be used to characterize the metabolite profiles of as many cultivars as possible for building a reference library. Preparative LC can be helpful to collect individual metabolite fractions and establish their identity. Several metabolites detected in 7 selected cultivars of *Curcuma* spp. by GC-MS and LC-MS analyses are reported first time in *Curcuma* spp. We suggest that the seven Indian cultivars of *Curcuma* spp. employed in our study can be used as sources of such compounds. High-throughput analysis of cultivar-specific and first-time detected compounds in the present study may lead to new drug candidates. The metabolites validated for their medicinal or other users can be quantified using simple techniques such as HPLC or TLC to ensure their presence in the herbal preparations.

## Data Availability

The datasets presented in this study can be found in online repositories. The names of the repository/repositories and accession number(s) can be found below: https://www.ebi.ac.uk/metabolights/MTBLS2790.

## Supplementary Material

Supplementary Figure 1
**(A)** Representative TIC chromatograms from negative ion (−) ESI-HPLC from cultivars (A) Alleppey Supreme, (B) Duggirala Red, (C) Prathibha, (D) Salem, (E) Suguna of *Curcuma longa* L. and cvs. (F) Kasturi Araku, (G) Kasturi Avidi of *C. aromatica* Salisb. Peak labelled IS represents internal standard. **(B)** Representative TIC chromatograms from positive ion (+) ESI-HPLC from cultivars (A) Alleppey Supreme, (B) Duggirala Red, (C) Prathibha, (D) Salem, (E) Suguna of *Curcuma longa* L. and cvs. (F) Kasturi Araku, (G) Kasturi Avidi of *C. aromatica* Salisb. Peak labelled IS represents internal standard.

Supplementary Figure 2 MS and MS/MS spectra of cultivar-specific compounds first time reported from genus *Curcuma*, identified from cultivars of *Curcuma longa* L. and *C. aromatica* Salisb. detected in essential oil (1–35) and rhizome extracts (36–39) by GC-MS (MS spectra) and LC-MS (MS/MS spectra) respectively. (Spectra 1–23 corresponds to panel numbers: 1–23 of Figure 2 and serial numbers: 1–23 of Table 2. Spectra 24–35 corresponds to panel numbers: 24–35 of Figure 2 and serial numbers: 1–12 of Table 3. Spectra 36 correspond to panel numbers 36 of Figure 2 and serial number 1 of Table 6. Spectra 37–39 correspond to panel number 37–39 of Figure 2 and serial number 1–3 of Table 7). (1) 1,2-Cyclohexanediol, 1-methyl-4-(1-methylethyl)-, (2) trans, trans-Octa-2,4-dienyl acetate, (3) Phenol, 2-methoxy-3-(2- propenyl)-, (4) 3-Isopropyl-4-methyl-1-pentyn-3-ol (5) 5,9-Tetradecadiyne, (6) Naphthalene, 5-butyl-1,2,3,4-tetrahydro-, (7) Santolina alcohol, (8) 2-Pentanone, 4-mercapto-4-methyl-, (9) 8-Methylene-3-oxatricyclo[5.2.0.0(2,4)]nonane, (10) 7-Tetracyclo[6.2.1.0(3.8)0(3.9)]undecanol, 4,4,11,11 tetramethyl-, (11) Bicyclo[2.2.1]hept-2-ene, 2,3-dimethyl-, (12) 1H-3a,7-Methanoazulene, 2,3,4,7,8,8a-hexahydro-3,6,8,8-tetramethyl-, [3R-(3à,3aá, 7á, 8aà)]-, (13) Cholesta-8,24-dien-3-ol, 4-methyl-, (3á,4à)-, (14) 4-Ethylphenethylamine, (15) Cyclohexanol, 2-methyl-5-(1-methylethenyl)-, (16) Cyclohexane, 1,2-dimethyl-3,5-bis(1-methylethenyl)-, (17) 5,8,11,14-Eicosatetraenoic acid, phenylmethyl ester, (all-Z)-, (18) 11-Dodecen-2-one, (19) E-11-Tetradecenoic acid, (20) 2-Nonen-4-yn-1-ol, (Z)-, (21) 3-Cyclohexen-1-one, 3,5,5-trimethyl-, (22) 6,10-Dodecadien-1-yn-3-ol, 3,7,11-trimethyl-, (23) 3-Octen-5-yne, 2,7-dimethyl-, (Z)-, (24) 1,3,5-Cycloheptatriene, (25) Bicyclo[3.1.0]hexane, 4-methyl-1-(1-methylethyl)-, didehydro deriv., (26) Bicyclo[3.2.1]oct-2-ene, 3-methyl-4-methylene-, (27) Oxirane, 2-(hexyn-1-yl)-3-methoxymethylene-, (28) Bergamotol, Z-α-trans-, (29) (1,3-Dimethyl-2-methylene-cyclopentyl)-methanol, (30) 12-Oxabicyclo[9.1.0]dodeca-3,7-diene, 1,5,5,8-tetramethyl-, [1R-(1R*,3E,7E,11R*)]-, (31) Isolongifolene, 4,5,9,10-dehydro-, (32) Z,Z,Z-4,6,9-Nonadecatriene, (33) 6-(p-Tolyl)-2-methyl-2-heptenol, (34) 6-Tridecen-4-yne, (Z)-, (35) 1,4-Cyclohexadiene, 1-methyl-, (36) Kaempferol-3,7-O-dimethyl ether, (37) 5,7,8-Trihydroxy-2′,5′-dimethoxy-3′,4′-methylene dioxyisoflavanone, (38) Chavicol, (39) Kaempferol-3-O-rutinoside-7-O-glucoside.

Supplementary Table 1 A list of secondary metabolites identified by GC-MS in the essential oil from the rhizomes of five cvs. of *C. longa* L. and two cvs. of *C. aromatica* Salisb. Abbreviations: AS, Alleppey Supreme; DR, Duggirala Red; PR, Prathibha; SA, Salem; SU, Suguna; KAr, Kasturi Araku; KAv, Kasturi Avidi.

Supplementary Table 2 A list of secondary metabolites identified by LC-MS in the rhizomes extract of five cvs. of *C. longa* L. and two cvs. of *C. aromatica* Salisb. Abbreviations used: AS, Alleppey Supreme; DR, Duggirala Red; PR, Prathibha; SA, Salem; SU, Suguna; KAr, Kasturi Araku; KAv, Kasturi Avidi.

Supplementary Table 3 Identification parameters of first-time reported compounds by GC-MS in the essential oil from the seven cultivars of *Curcuma* spp. along with the methods used in previous literature.

Supplementary Table 4 Identification parameters of first-time reported compounds by LC-MS in the rhizome extracts of genus *Curcuma* along with the methods used in related previous literature.

Supplementary Table 5 The list of 80 compounds identified by GCMS analysis.

Supplementary Table 6 The list of 62 compounds identified by LCMS analysis.

## Abbreviations

CU, curcumin; cvs, cultivars; DMC, demethoxy curcumin; BDMC, bisdemethoxycurcumin.
