# Supplementary material for: Variable Secondary Metabolite Profiles Across Cultivars of Curcuma longa L. and C. aromatica Salisb
Source: Front Pharmacol. 2021 Jun 30;12:659546. doi: 10.3389/fphar.2021.659546 (PMC8278146; doi:10.3389/fphar.2021.659546)

Supplementary Material

Supplementary Figure Legends

Supplementary Figure S1 A. Representative TIC chromatograms from negative ion (-) ESI-HPLC from cultivars (A) Alleppey Supreme, (B) Duggirala Red, (C) Prathibha, (D) Salem, (E) Suguna of *Curcuma longa* L. and cvs. (F) Kasturi Araku, (G) Kasturi Avidi of *C. aromatica* Salisb. Peak labelled IS represents internal standard.

Supplementary Figure S1 B. Representative TIC chromatograms from positive ion (+) ESI-HPLC from cultivars (A) Alleppey Supreme, (B) Duggirala Red, (C) Prathibha, (D) Salem, (E) Suguna of *Curcuma longa* L. and cvs. (F) Kasturi Araku, (G) Kasturi Avidi of *C. aromatica* Salisb. Peak labelled IS represents internal standard.

Supplementary Figure S2. MS and MS/MS spectra of cultivar-specific compounds first time reported from genus *Curcuma*, identified from cultivars of *Curcuma longa* L. and *C. aromatica* Salisb. detected in essential oil (1-35) and rhizome extracts (36-39) by GC-MS (MS spectra) and LC-MS (MS/MS spectra) respectively. (Spectra 1-23 corresponds to panel numbers: 1-23 of Figure 2 and serial numbers: 1-23 of Table 2. Spectra 24-35 corresponds to panel numbers: 24-35 of Figure 2 and serial numbers: 1-12 of Table 3. Spectra 36 correspond to panel numbers 36 of Figure 2 and serial number 1 of Table 6. Spectra 37-39 correspond to panel number 37-39 of Figure 2 and serial number 1-3 of Table 7).

(1) 1,2-Cyclohexanediol, 1-methyl-4-(1-methylethyl)-, (2) trans, trans-Octa-2,4-dienyl acetate, (3) Phenol, 2-methoxy-3-(2- propenyl)-, (4) 3-Isopropyl-4-methyl-1-pentyn-3-ol (5) 5,9-Tetradecadiyne, (6) Naphthalene, 5-butyl-1,2,3,4-tetrahydro-, (7) Santolina alcohol, (8) 2-Pentanone, 4-mercapto-4-methyl-, (9) 8-Methylene-3-oxatricyclo[5.2.0.0(2,4)]nonane, (10) 7-Tetracyclo[6.2.1.0(3.8)0(3.9)]undecanol, 4,4,11,11 tetramethyl-, (11) Bicyclo[2.2.1]hept-2-ene, 2,3-dimethyl-, (12) 1H-3a,7-Methanoazulene, 2,3,4,7,8,8a-hexahydro-3,6,8,8-tetramethyl-, [3R-(3à,3aá,7á,8aà)]-, (13) Cholesta-8,24-dien-3-ol, 4-methyl-, (3á,4à)-, (14) 4-Ethylphenethylamine, (15) Cyclohexanol, 2-methyl-5-(1-methylethenyl)-, (16) Cyclohexane, 1,2-dimethyl-3,5-bis(1-methylethenyl)-, (17) 5,8,11,14-Eicosatetraenoic acid, phenylmethyl ester, (all-Z)-, (18) 11-Dodecen-2-one, (19) E-11-Tetradecenoic acid, (20) 2-Nonen-4-yn-1-ol, (Z)-, (21) 3-Cyclohexen-1-one, 3,5,5-trimethyl-, (22) 6,10-Dodecadien-1-yn-3-ol, 3,7,11-trimethyl-, (23) 3-Octen-5-yne, 2,7-dimethyl-, (Z)-, (24) 1,3,5-Cycloheptatriene, (25) Bicyclo[3.1.0]hexane, 4-methyl-1-(1-methylethyl)-, didehydro deriv., (26) Bicyclo[3.2.1]oct-2-ene, 3-methyl-4-methylene-, (27) Oxirane, 2-(hexyn-1-yl)-3-methoxymethylene-, (28) Bergamotol, Z-α-trans-, (29) (1,3-Dimethyl-2-methylene-cyclopentyl)-methanol, (30) 12-Oxabicyclo[9.1.0]dodeca-3,7-diene, 1,5,5,8-tetramethyl-, [1R-(1R*,3E,7E,11R*)]-, (31) Isolongifolene, 4,5,9,10-dehydro-, (32) Z,Z,Z-4,6,9-Nonadecatriene, (33) 6-(p-Tolyl)-2-methyl-2-heptenol, (34) 6-Tridecen-4-yne, (Z)-, (35) 1,4-Cyclohexadiene, 1-methyl-, (36) Kaempferol-3,7-O-dimethyl ether, (37) 5,7,8-Trihydroxy-2′,5′-dimethoxy-3′,4′-methylene dioxyisoflavanone, (38) Chavicol, (39) Kaempferol-3-O-rutinoside-7-O-glucoside.

Supplementary Figure S1 A

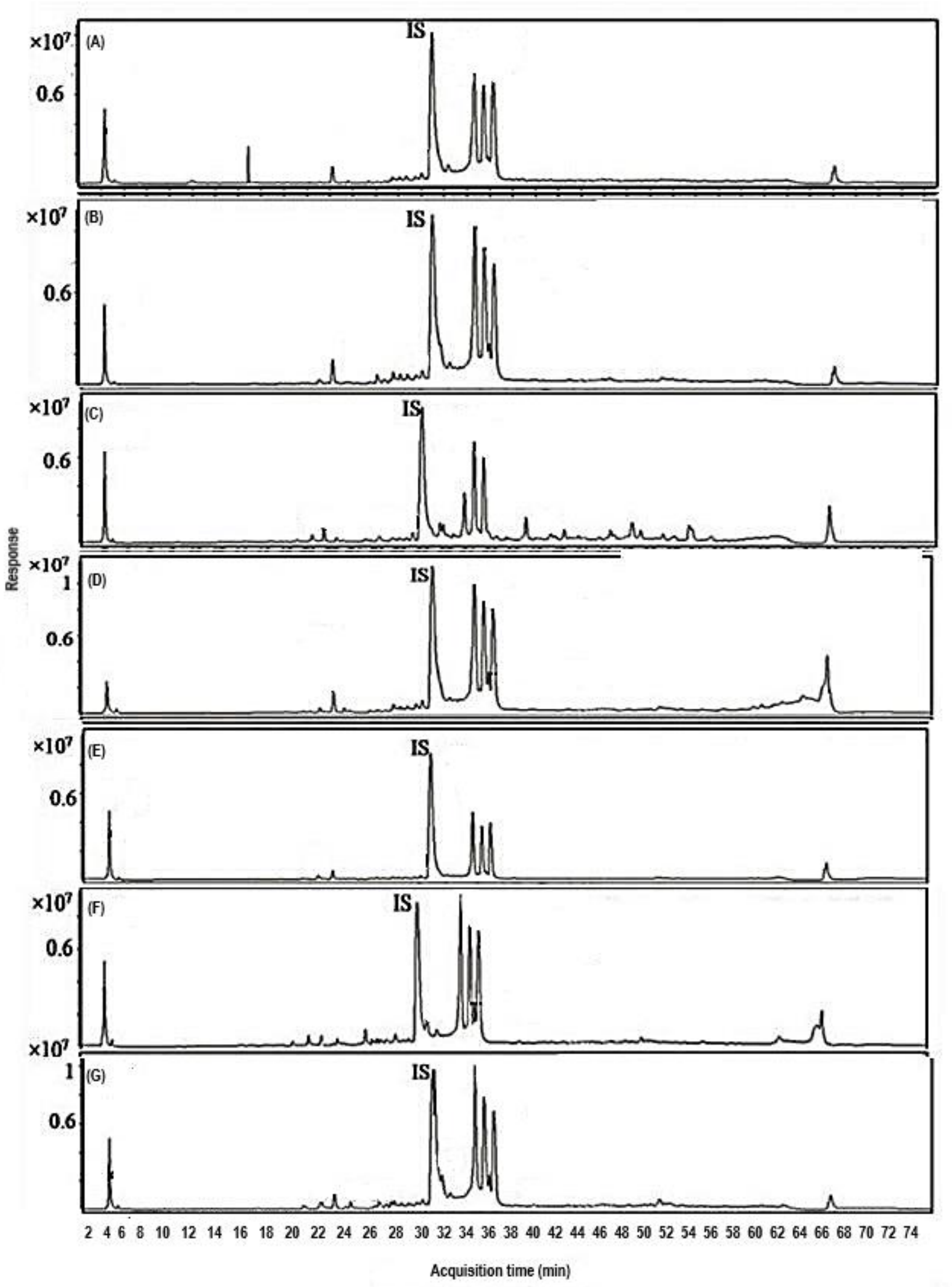

Supplementary Figure S1 B

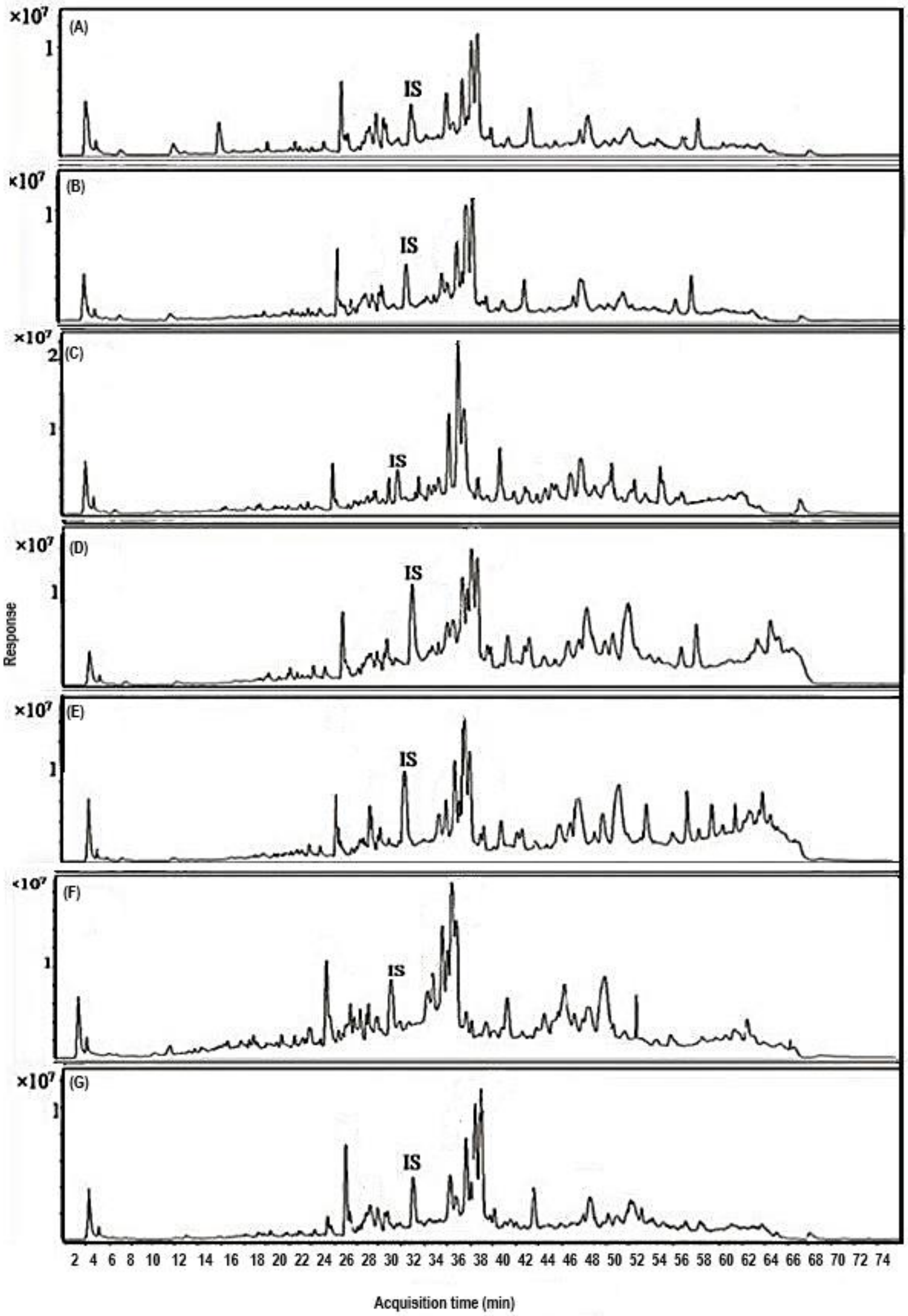

Supplement: Supplementary file 1 [file Image1.pdf]
