# Supplementary figures and images for: Variable Secondary Metabolite Profiles Across Cultivars of Curcuma longa L. and C. aromatica Salisb
Source: Front Pharmacol. 2021 Jun 30;12:659546. doi: 10.3389/fphar.2021.659546 (PMC8278146; doi:10.3389/fphar.2021.659546)

## Supplementary Figure S2

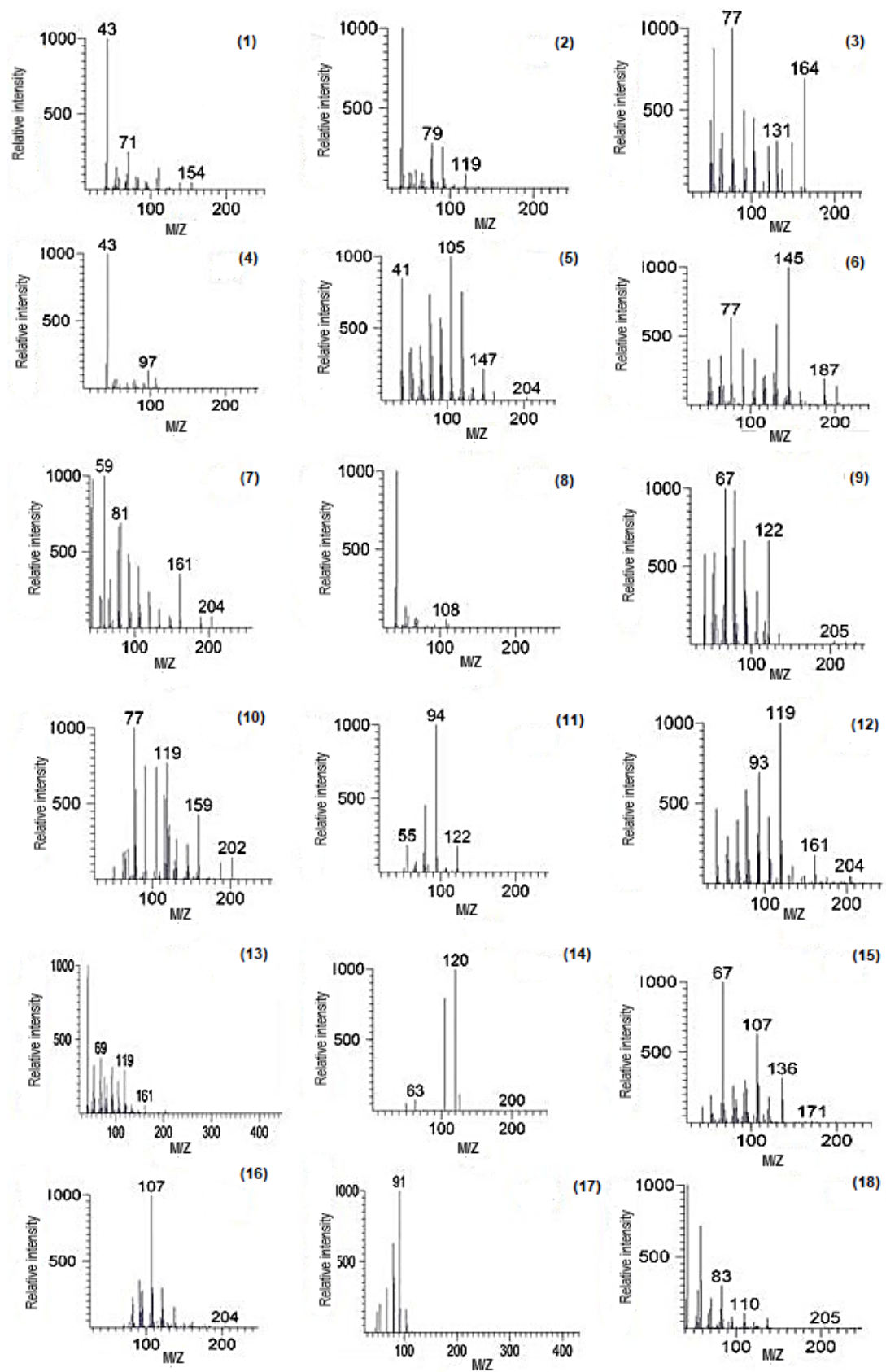

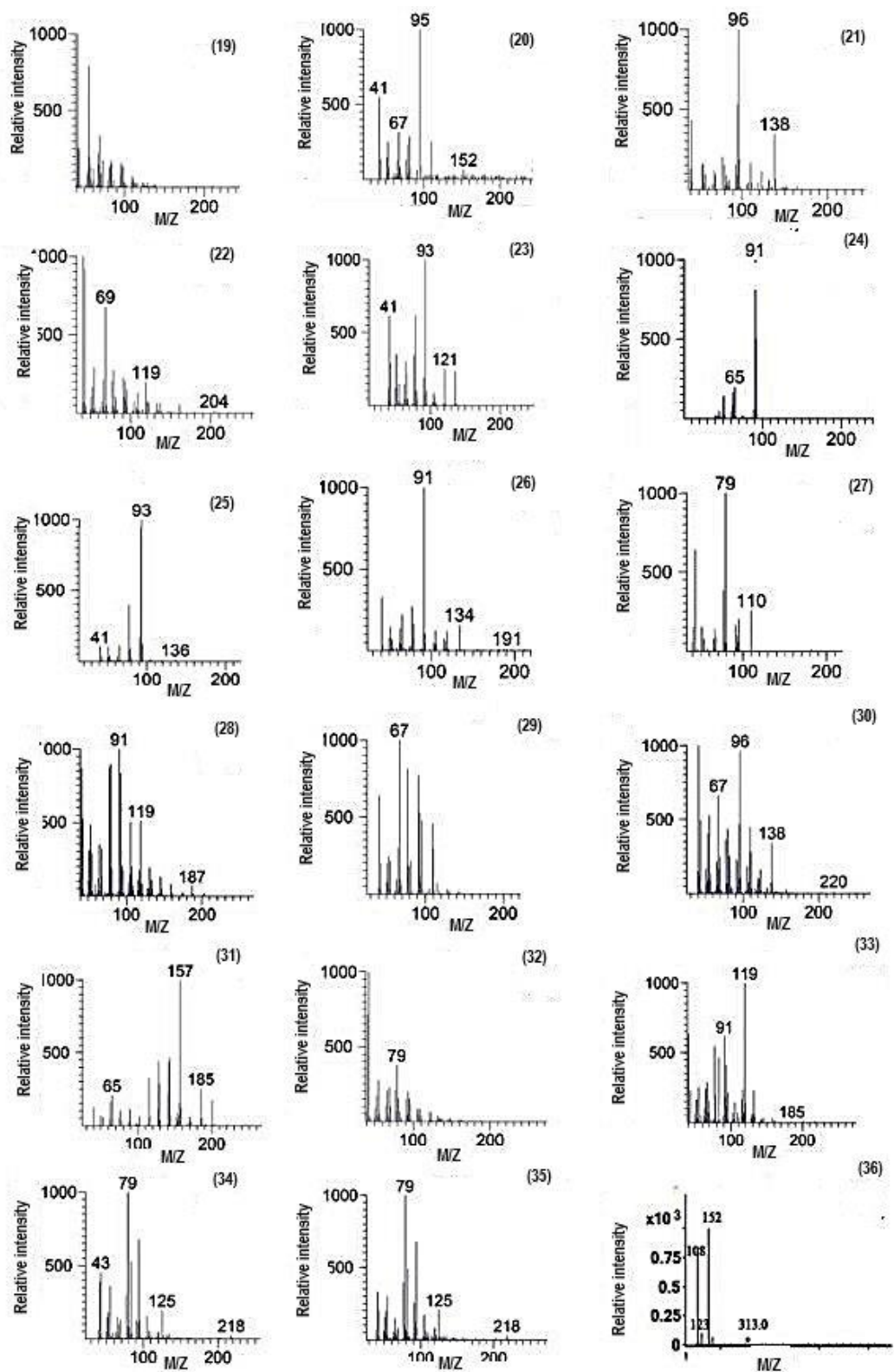

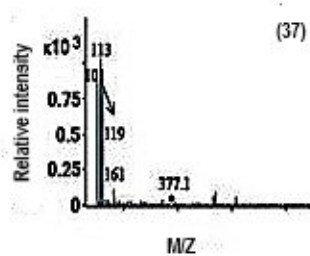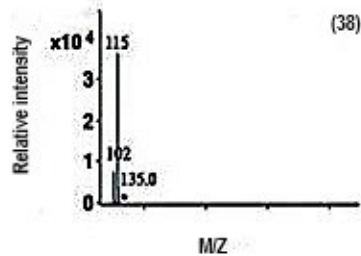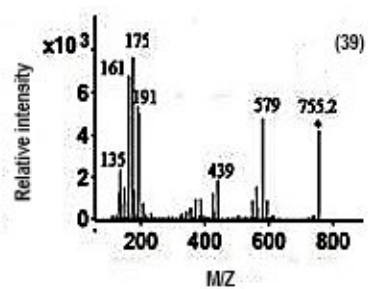

Supplement: Supplementary file 2 [file Image2.pdf]
