# Supplementary material for: Variable Secondary Metabolite Profiles Across Cultivars of Curcuma longa L. and C. aromatica Salisb
Source: Front Pharmacol. 2021 Jun 30;12:659546. doi: 10.3389/fphar.2021.659546 (PMC8278146; doi:10.3389/fphar.2021.659546)
Supplement: Supplementary file 3 [file Table1.docx]

**Supplementary Table S1**. A list of secondary metabolites identified by GC-MS in the essential oil from the rhizomes of five cvs. of *C. longa* L. and two cvs. of *C. aromatica*Salisb. Abbreviations used: AS: Alleppey Supreme; DR: Duggirala Red; PR: Prathibha; SA: Salem; SU: Suguna; KAr: Kasturi Araku; and KAv: Kasturi Avidi.

| **Sl.**  **No.** | **RT (Min)** | **Compound name** | ***C. longa* L.**  **(cultivars)** | | | | | ***C. aromatica* Salisb.**  **(cultivars)** | |
| --- | --- | --- | --- | --- | --- | --- | --- | --- | --- |
|  |  |  | **AS** | **DR** | **PR** | **SA** | **SU** | **KAr** | **KAv** |
| 1 | 2.16 | 1,3,5-Cycloheptatriene | + | - | + | + | + | + | + |
| 2 | 4.13 | α-Thujene | + | + | + | + | + | + | + |
| 3 | 4.27 | 1s-α-Pinene | + | + | + | + | + | + | + |
| 4 | 4.56 | Camphene | + | + | + | - | + | - | - |
| 5 | 5.05 | Sabinene | + | + | + | + | + | + | + |
| 6 | 5.32 | 2-Pentanone, 4-mercapto-4-methyl- | - | - | + | - | - | - | - |
| 7 | 5.39 | 5,8,11,14-Eicosatetraenoic acid, phenylmethyl ester, (all-Z)- | - | - | - | + | - | - | - |
| 8 | 5.63 | Bicyclo[4.1.0]hept-2-ene, 3,7,7-trimethyl- | - | **+** | - | - | - | - | + |
| 9 | 5.70 | Bicyclo[3.1.0]hexane, 4-methyl-1-(1-methylethyl)-, didehydro deriv. | - | + | + | + | + |  | - |
| 10 | 5.72 | α-Phellandrene | + | + | - | + | + | + | - |
| 11 | 6.00 | α-Terpinene | + | + | - | + | - | + | + |
| 12 | 6.18 | β or m-Cymene | + | + | + | + | + | + | + |
| 13 | 6.27 | Limonene | + | - | - | - | + | + | + |
| 14 | 6.34 | Eucalyptol | - | - | - | - | - | + | - |
| 15 | 6.38 | 1,2-Cyclohexanediol, 1-methyl-4-(1-methylethyl)- | + | - | - | - | - | - | - |
| 16 | 6.74 | cis-Ocimene | - | + | + | + | + | + | + |
| 17 | 7.01 | γ-Terpinene | + | + | + | + | + | + | - |
| 18 | 7.82 | Terpinolene | + | + | + | + | + | + | + |
| 19 | 7.91 | 3-Octen-5-yne, 2,7-dimethyl-, (Z)- | - | - | - | - | - | - | + |
| 20 | 8.15 | Linalool | + | + | + | - | + | - | - |
| 21 | 8.22 | trans, trans-Octa-2,4-dienyl acetate | + | - | - | - | - | - | - |
| 22 | 9.35 | bicyclo[3.2.1]oct-2-ene, 3-methyl-4-methylene | - | + | - | - | + | - | + |
| 23 | 9.68 | Camphor | - | - | + | - | **-** | - | - |
| 24 | 9.75 | Oxirane, 2-(hexyn-1-yl)-3-methoxymethylene- | - | + | + | - | - | + | + |
| 25 | 10.16 | Isoborneol | - | - | + | - | **-** | - | - |
| 26 | 10.68 | 2-Nonen-4-yn-1-ol, (Z)- | - | - | - | - | + | - | - |
| 27 | 10.81 | Citral | - | - | - | - | - | + | - |
| 28 | 10.83 | Terpinene-4-ol | + | + | + | + | + | - | - |
| 29 | 11.08 | p-Cymen-8-ol | - | - | - | - | - | + | - |
| 30 | 11.25 | α-Terpineol | + | - | + | + | + | + | + |
| 31 | 11.72 | 8-Methylene-3-oxatricyclo[5.2.0.0(2,4)]nonane | - | - | + | - | - | - | - |
| 32 | 13.59 | 3-Isopropyl-4-methyl-1-pentyn-3-ol | - | + | - | - | - | - | - |
| 33 | 15.46 | Carvacrol | + | - | - | - | - | - | - |
| 34 | 17.05 | Phenol, 2-methoxy-3-(2- propenyl)- | + | - | - | - | - | - | - |
| 35 | 17.87 | β-Elemene | - | - | + | - | - | - | - |
| 36 | 18.86 | trans-α-Bergamotene | + | + | + | + | + | + | + |
| 37 | 19.15 | α-Santalene | - | - | + | - | - | - | - |
| 38 | 19.16 | 7-Tetracyclo[6.2.1.0(3.8)0(3.9)]undecanol, 4,4,11,11 tetramethyl- | - | - | + | - | - | - | - |
| 39 | 19.23 | α-Caryophyllene | + | + | + | + | + | + | + |
| 40 | 19.37 | trans-β-Farnesene | + | + | + | + | + | + | + |
| 41 | 19.41 | Bicyclo[2.2.1]hept-2-ene, 2,3-dimethyl- | - | - | + | - | - | - | - |
| 42 | 19.45 | 5,9-Tetradecadiyne | - | + | - | - | - | - | - |
| 43 | 19.69 | 1H-3a,7-Methanoazulene, 2,3,4,7,8,8a-hexahydro-3,6,8,8-tetramethyl-, [3R-(3à,3aá,7á,8aà)]- | - | - | + | - | - | - | - |
| 44 | 19.81 | Sabinene hydrate | - | - | - | - | - | - | + |
| 45 | 19.88 | Ar-Curcumene | + | + | + | + | + | + | + |
| 46 | 20.25 | α-Zingiberene | + | + | + | + | + | + | + |
| 47 | 20.40 | cis-α-Bisabolene | - | - | + | - | - | + | - |
| 48 | 20.62 | Naphthalene, 5-butyl-1,2,3,4-tetrahydro- | - | + | - | - | - | - | - |
| 49 | 20.83 | β-Sesquiphellandren | - | + | - | + | + | - | + |
| 50 | 20.90 | Bergamotol, Z-α-trans- | + | - | - | + | - | - | - |
| 51 | 20.92 | Neoisolongifolene, 8,9-dehydro- | - | - | - | - | - | + | - |
| 52 | 21.05 | (1,3-Dimethyl-2-methylene-cyclopentyl)-methanol | + | + | - | + | + | - | + |
| 53 | 21.56 | Cholesta-8,24-dien-3-ol, 4-methyl-, (3á,4à)- | - | - | + | - | - | - | - |
| 54 | 21.67 | 12-Oxabicyclo[9.1.0]dodeca-3,7-diene, 1,5,5,8-tetramethyl-, [1R-(1R*,3E,7E,11R*)]- | - | + | - | - | - | + | - |
| 55 | 21.68 | Aromadendrene | - | - | + | - | - | - | - |
| 56 | 21.79 | Nerolidol | - | + | + | + | + | - | + |
| 57 | 21.81 | 3-Cyclohexen-1-one, 3,5,5-trimethyl- | - | - | - | - | + | - | - |
| 58 | 21.85 | Caryophyllene oxide | - | - | + | - | - | - | - |
| 59 | 22.10 | Isolongifolene, 4,5,9,10-dehydro- | + | + | - | + | + | + | - |
| 60 | 22.16 | Tumerone | + | + | + | + | + | + | + |
| 61 | 22.26 | 1,3,5-Cycloheptatriene, 3,7,7-trimethyl- | + | - | - | - | + | - | + |
| 62 | 22.33 | Z,Z,Z-4,6,9-Nonadecatriene | - | + | - | - | - | - | + |
| 63 | 22.60 | α-Bisabolol | - | - | + | - | - | - | - |
| 64 | 22.98 | 6-(p-Tolyl)-2-methyl-2-heptenol | + | - | - | - | + | - | + |
| 65 | 22.99 | α-Elemenone | - | - | + | - | - | - | - |
| 66 | 23.14 | 6-Tridecen-4-yne, (Z)- | - | + | + | - | + | - | - |
| 67 | 23.15 | 1,4-Cyclohexadiene, 1-methyl- | - | - | - | - | - | + | + |
| 68 | 23.27 | 6,10-Dodecadien-1-yn-3-ol, 3,7,11-trimethyl- | - | - | - | - | + | - | - |
| 69 | 23.63 | Santolina alcohol | - | + | - | - | - | - | - |
| 70 | 25.87 | Ar-Tumerone | - | + | - | + | - | - | + |
| 71 | 25.88 | 4-Ethylphenethylamine | - | - | + | - | - | - | - |
| 72 | 25.95 | endo-Borneol | - | - | + | - | - | - | - |
| 73 | 26.57 | Cyclohexanol, 2-methyl-5-(1-methylethenyl)- | - | - | + | - | - | - | - |
| 74 | 26.59 | Cyclohexane, 1,2-dimethyl-3,5-bis(1-methylethenyl)- | - | - | + | - | - | - | - |
| 75 | 27.34 | Curlone | + | + | + | + | + | + | + |
| 76 | 36.82 | 2-Tridecanone | - | - | + | - | - | - | - |
| 77 | 36.83 | 11-Dodecen-2-one | - | - | - | + | - | - | - |
| 78 | 37.15 | E-11-Tetradecenoic acid | - | - | - | + | - | - | - |
| 79 | 37.82 | Nonanoic acid | - | - | - | - | + | - | - |
| 80 | 39.17 | 2-Heptadecanone | + | + | + | + | + | + | + |
