# Supplementary material for: Variable Secondary Metabolite Profiles Across Cultivars of Curcuma longa L. and C. aromatica Salisb
Source: Front Pharmacol. 2021 Jun 30;12:659546. doi: 10.3389/fphar.2021.659546 (PMC8278146; doi:10.3389/fphar.2021.659546)
Supplement: Supplementary file 4 [file Table2.docx]

**Supplementary Table S2**. A list of secondary metabolites identified by LC-MS in the rhizomes extract of five cvs. of *C. longa* L. and two cvs. of *C. aromatica*Salisb. Abbreviations used: AS: Alleppey Supreme; DR: Duggirala Red; PR: Prathibha; SA: Salem; SU: Suguna; KAr: Kasturi Araku; and KAv: Kasturi Avidi.

| **Sl.**  **No.** | **RT (Min)** | **Compound name** | ***C. longa***  **(cultivars)** | | | | | ***C. aromatica***  **(cultivars)** | |
| --- | --- | --- | --- | --- | --- | --- | --- | --- | --- |
|  |  |  | **AS** | **DR** | **PR** | **SA** | **SU** | **KAr** | **KAv** |
| 1 | 2.2 | 5,7,8-Trihydroxy-2′,5′-dimethoxy-3′,4′-methylene dioxyisoflavanone | + | + | - | - | - | - | + |
| 2 | 2.49 | 1,7-Diphenyl-1,6-heptadiene-3,5-dione | + | - | - | + | - | - | - |
| 3 | 10.76 | Kaempferol-3-rhamnoside | + | + | - | - | - | - | - |
| 4 | 11.1 | 1,2,3,4-Tetraphenylbutane-2,3-diol | - | - | - | - | - | + | - |
| 5 | 12.77 | Kaempferol-3,7-O-dimethyl ether | + | - | - | - | - | - | - |
| 6 | 16.8 | Hydroferulic acid | - | - | - | - | - | + | - |
| 7 | 21.2 | 1-Hepten-3-one, 5-hydroxy-1,7-bis(3,4-dihydroxyphenyl)- | - | - | + | + | + | + | + |
| 8 | 22.16 | 4-(*p*-Hydroxyphenyl)-3-buten-2-one | + | + | - | + | - | - | + |
| 9 | 22.8 | 1,7-Bis(4-hydroxyphenyl)-3,5-heptanediol | - | - | - | - | - | + | - |
| 10 | 23.11 | 5-Hydroxy-7-(4-hydroxyphenyl)-1-phenyl-(1E)-1-heptene | + | + | + | + | - | + | + |
| 11 | 23.5 | 1,5-Bis(4-hydroxy-3-methoxyphenyl)-1,4-pentadien-3-one | + | + | + | + | + | + | + |
| 12 | 24.7 | Ar-Turmerone | + | + | + | + | + | + | + |
| 13 | 25.2 | 4-Hepten-3-one, 5-hydroxy-1,7-bis(4-hydroxyphenyl)- | - | - | + | - | - | - | - |
| 14 | 25.4 | Tetrahydroxybisdemethoxycurcumin | + | + | + | + | + | + | + |
| 15 | 25.36 | Turmeronol | + | + | - | - | + | - | - |
| 16 | 25.8 | 1-(4-Hydroxy-3-methoxyphenyl)-7-(4-hydroxy-3,5-dimethoxypheny)-4,6-heptadiene-3-one | + | + | - | + | - | + | + |
| 17 | 25.9 | Tetrahydrodemethoxycurcumin | + | + | + | + | + | + | + |
| 18 | 26.0 | 1,5-Bis(3,4-methylenedioxyphenyl)-1,4-pentadien-3-one | - | - | - | - | + | - | + |
| 19 | 26.1 | 1-(4-Hydroxyphenyl)-7-(4-hydroxy-3-methoxyphenyl)-1,4,6-heptatrien-3-one | + | + | + | + | + | + | + |
| 20 | 26.3 | 1,7-Bis(4-hydroxy-3-methoxyphenyl)-1,4,6-heptatrien-3-one | + | + | + | + | + | + | + |
| 21 | 26.5 | Tetrahydroxycurcumin | + | + | + | + | + | + | + |
| 22 | 26.7 | 1-Hydroxy-1-(3,4-dihydroxyphenyl)-7-(4-hydroxy-3-methoxyphenyl)-6-hepten-3,5-dione | + | - | - | - | - | + | + |
| 23 | 26.8 | 5,7-Dihydroxy-2-(4-hydroxyphenyl)-chroman-4-one | - | - | + | - | - | - | - |
| 24 | 26.84 | 1-(4-Hydroxy-3-methoxyphenyl)-7-(4-hydroxy-3,5-dimethoxyphenyl)-1,4,6-heptatrien-3-one | + | + | + | + | + | + | + |
| 25 | 27.4 | 1,7-Bis(4-hydroxyphenyl)-1-heptene-3,5-dione | + | + | + | + | + | - | + |
| 26 | 28.8 | 1-(4-Hydroxy-3-methoxyphenyl)-5-(4-hydroxyphenyl)-1,4-pentadiene-3-one pentadiene-3-one | - | - | - | - | - | - | + |
| 27 | 29.47 | Tetradecanoic acid/myristic acid | + | - | - | - | - | - | - |
| 28 | 30.0 | 1,7-Bis(4-hydroxyphenyl)-1,4,6-heptatrien-3-one | + | + | + | + | + | - | + |
| 29 | 31.6 | 1,6-Heptadiene-3,5-dione, 1-(3,4-dihydroxyphenyl)-7-(4-hydroxy phenyl)- | + | + | + | + | + | + | + |
| 30 | 31.8 | 7-(4-Hydroxy-3-methoxyphenyl)-1-(4-hydroxy phenyl)-4,6-heptadien-3-one | + | - | - | - | - | - | + |
| 31 | 32.0 | 1-(4-Hydroxy-3-methoxyphenyl)-7-(4-hydroxy-3,5-dimethoxypheny)-4,6-heptadiene-3-one | - | - | + | - | - | - | - |
| 32 | 32.3 | 1-(3,4-Dihydroxyphenyl)-7-(4-hydroxy-3-methoxyphenyl)-hepta-1,6-diene-3,5-dione | + | + | + | + | + | + | + |
| 33 | 32.9 | 1,7-Bis(4-hydroxy-3,5-dimethoxyphenyl)-1,6-heptadiene-3,5-dione | - | - | + | - | - | - | - |
| 34 | 33.2 | Kaempferol-3-O-rutinoside-7-O-glucoside | - | - | + | + | - | - | - |
| 35 | 34.36 | Coumaran | + | + | - | + | + | - | - |
| 36 | 34.40 | Methyl-7-methoxycoumarin,4- | - | - | - | - | + | - | - |
| 37 | 34.5 | Bisdemthoxycurcumin | + | + | + | + | + | + | + |
| 38 | 34.57 | 5,7-Dihydroxy-4-methylcoumarin | + | - | - | + | + | - | + |
| 39 | 34.65 | 3-Acetyl coumarin | + | - | + | + | + | - | + |
| 40 | 35.1 | Hydrocinnamic acid | - | - | - | - | + | + | - |
| 41 | 35.3 | Tumerone | + | - | - | - | - | - | - |
| 42 | 35.4 | Demethoxycurcumin | + | + | + | + | + | + | + |
| 43 | 35.56 | 5’-Methoxycurcumin | - | - | + | - | - | - | - |
| 44 | 35.8 | Dihydrocurcumin | + | + | + | + | + | + | + |
| 45 | 35.82 | 1,7-Bis(3,4,5-trimethoxyphenyl)-l,6-heptadiene-3,5-dione | - | - | + | - | + | + | + |
| 46 | 35.99 | 7-(3,4-Dihydroxyphenyl)-5-hydroxy-1-phenyl-(1E)-1-heptene | + | - | - | - | - | - | + |
| 47 | 36.2 | Curcumin | + | + | + | + | + | + | + |
| 48 | 36.67 | Curcumenol | + | - | - | + | + | + | + |
| 49 | 38.3 | 1,7-Bis(3,4-dimethoxyphenyl)-1,6-heptadiene-3,5-dione | - | - | + | - | - | - | - |
| 50 | 38.83 | Chavicol | + | - | - | - | + | - | - |
| 51 | 39.8 | (-)-(12E,2S,3S,4R, 5R,6R, 9S,11S, 15R)-3,15-Dibenzoyloxy-5,6-epoxylathyr-12-en-14-one | - | - | - | - | - | - | + |
| 52 | 40.2 | (6S)-2-Methyl-6-[(1R,5S)-(4-methene-5-hydroxyl-2-cyclohexen)-2-hepten-4-one | - | - | + | - | - | - | - |
| 53 | 42.2 | Luteolin-7-O-glucoside | + | - | - | - | - | - | - |
| 54 | 43.2 | 1-Heptene-3,5-dione, 1,7-bis-(4-hydroxy-3-methoxyphenyl)- | + | + | + | + | + | + | + |
| 55 | 44.6 | 4-Methylene-5-hydroxybisabola-2,10-diene-9-one | - | - | + | - | - | - | - |
| 56 | 46.50 | Curlone | + | + | + | - | + | + | + |
| 57 | 54.4 | 1,7-Bis(3,4-dimethoxyphenyl)-4,4-dimethyl-1,6-heptadiene-3,5-dione | - | - | + | - | - | - | - |
| 58 | 54.96 | 25-Benzylpentacyclo-(22.3.1.0.)-octacosa-1(27),3 (8),4,6,10(15),11,13,17(22), 18,20, 24(28), 25-dodecaen | + | - | - | - | - | - | - |
| 59 | 59.5 | Palmitic acid | - | - | - | + | - | - | - |
| 60 | 60.2 | Oleic acid | - | - | - | + | - | - | + |
| 61 | 60.6 | 1,7-Bis(3,5-diethyl-4-hydroxyphenyl)-1,6-heptadiene-3,5-dione | - | - | - | - | - | + | - |
| 62 | 63.8 | Stearic acid | - | - | - | + | - | - | - |
