# Supplementary material for: Variable Secondary Metabolite Profiles Across Cultivars of Curcuma longa L. and C. aromatica Salisb
Source: Front Pharmacol. 2021 Jun 30;12:659546. doi: 10.3389/fphar.2021.659546 (PMC8278146; doi:10.3389/fphar.2021.659546)
Supplement: Supplementary file 5 [file Table3.docx]

**Supplementary Table S3.** Identification parameters of first-time reported compounds by GC-MS in the essential oil from the seven cultivars of *Curcuma* spp. along with the methods used in previous literature.

| **Sl. nos.** | **Compound** | **Cultivar** | **Identification of the compounds done in present study** | | | | | **Identification of the compounds**  **done in previous literature** | |
| --- | --- | --- | --- | --- | --- | --- | --- | --- | --- |
|  |  |  | **Technique used** | **CAS Number in NIST library** | **Similarity of compound with NIST library**  **(**Software: Version 4.22 optimized for Pegasus®  **)** | **Mass** | **Mass fragmentation (m/z) pattern matched with NIST library** | **Technique used** | **Compound identification method** |
| 1 | 1,2-Cyclohexanediol, 1-methyl-4-(1-methylethyl)- | AS | GCMS | 33669-76-0 | 819 | 172.146 | 43, 71,111, 154 | GCMS | Matching NIST library data with reported literature and  Mass spectra |
| 2 | trans, trans-Octa-2,4-dienyl acetate | AS | GCMS | 30361-34-3 | 771 | 168.115 | 43,77,79 | GCMS | Compounds were confirmed by comparison with standard compounds and NIST library |
| 3 | Phenol, 2-methoxy-3-(2- propenyl)- | AS | GCMS | 1941-12-4 | 883 | 164.083 | 77,131,  164 | GCMS and Pyrolysis-GCMS | The pyrolysis products  were analyzed by GC–MS. The chromatographic peak area normalization method was used to calculate  groups the relative content of the points. |
| 4 | 3-Isopropyl-4-methyl-1-pentyn-3-ol | DR | GCMS | 5333-87-9 | 892 | 140.120 | 43,97,  107 | GCMS | Matching mass fragmentation pattern with those of compounds in NIST library |
| 5 | 5,9-Tetradecadiyne | DR | GCMS | 51255-61-9 | 771 | 190.172 | 41,105, 147 | GCMS | Comparison of the mass spectra and retention time with those reported in the literature |
| 6 | Naphthalene, 5-butyl-1,2,3,4-tetrahydro- | DR | GCMS | 66325-42-6 | 751 | 188.156 | 91,145,  188 | GCMS | Matching mass fragmentation pattern with those of compounds in NIST library |
| 7 | Santolina alcohol | DR | GCMS | 21149-19-9 | 744 | 154.135 | 59,81,  121 | GCMS | Retention indices and comparison of mass spectral fragmentation pattern with reported literature |
| 8 | 2-Pentanone, 4-mercapto-4-methyl- | PR | GCMS | 19872-52-7 | 900 | 132.060 | 43,55,  132 | GCMS | RI, mass spectrum and odor quality with those of an authentic compound |
| 9 | 8-Methylene-3-oxatricyclo[5.2.0.0(2,4)]nonane | PR | GCMS | PubChem CID:  556437 | 759 | 136.08 | 40,79, 92 | GCMS | Comparison of mass spectra with NIST 2005a libraries as well as by comparison of retention times |
| 10 | 7-Tetracyclo[6.2.1.0(3.8)0(3.9)]undecanol, 4,4,11,11 tetramethyl- | PR | GCMS | 74842-43-6 | 703 | 220.182 | 77,119,  159 | GCMS | Comparison of retention indices, using an online natural products library |
| 11 | Bicyclo[2.2.1]hept-2-ene, 2,3-dimethyl- | PR | GCMS | 529-16-8 | 891 | 122.109 | 79,94,  122 | GCMS | Comparison of the retention times with authentic substances and mass spectra with NIST, Willy library data of the GCMS system and literature data |
| 12 | 1H-3a,7-Methanoazulene, 2,3,4,7,8,8a-hexahydro-3,6,8,8-tetramethyl-, [3R-(3à,3aá,7á,8aà)]- | PR | GCMS | 469-61-4 | 805 | 204.187 | 93,119,  161 | GCMS | Retention indices method |
| 13 | Cholesta-8,24-dien-3-ol, 4-methyl-, (3á,4à)- | PR | GCMS | 7199-92-0 | 804 | 398.354 | 69,105,  119 | GCMS | Identified by similarity searches in the NIST and Mass Spectral Wiley database library |
| 14 | 4-Ethylphenethylamine | PR | GCMS | 64353-29-3 | 946 | 149.120 | 63,120 | GCMS | Identified on the basis of retention indices and by comparison of their mass spectral patterns (NIST 08.L database/chemstation data system) with previously reported in literature. |
| 15 | Cyclohexanol, 2-methyl-5-(1-methylethenyl)- | PR | GCMS | 619-01-2 | 729 | 154.135 | 67,107,  136 | GCMS | Identification was based on retention time. |
| 16 | Cyclohexane, 1,2-dimethyl-3,5-bis(1-methylethenyl)- | PR | GCMS | 62337-99-9 | 704 | 192.187 | 107,149 | GCMS | Identified on the basis of RI and comparing mass spectral fragmentation patterns with those reported in literatureand stored on the MS library (NIST database) |
| 17 | 5,8,11,14-Eicosatetraenoic acid, phenylmethyl ester, (all-Z)- | SA | GCMS | 77509-05-8 | 723 | 394.287 | 67,91,  205 | GCMS | NIST library was used for identification of the compounds |
| 18 | 11-Dodecen-2-one | SA | GCMS | 5009-33-6 | 857 | 182.167 | 43,124,  182 | GCMS | Data analysis was carried out by using the EPA/NIH/ MASS database (NBS library database). Retention times and mass spectra of compounds were compared with those of authentic compounds. |
| 19 | E-11-Tetradecenoic acid | SA | GCMS | PubChem CID:  5362745 | 912 | 226.193 | 41,55,69 | GCMS | Compounds were compared with NIST library data of the peak with those reported in literature, mass spectra of the peak with literature data. |
| 20 | 2-Nonen-4-yn-1-ol, (Z)- | SU | GCMS | 134225-90-4 | 867 | 154.135 | 41, 67,  95, 138 | GCMS | The components were identified by comparing relative retention time and mass spectra with those of standards, FFNSC 1.2 library data of the GCMS system and literature data. Relative retention indices was used for further confirmation of compounds. |
| 21 | 3-Cyclohexen-1-one, 3,5,5-trimethyl- | SU | GCMS | 471-01-2 | 746 | 138.104 | 96, 138 | SPME-GCMS | The mass spectra of the compounds were identified by comparison with spectra in Wiley 6N and NIST98 libraries. |
| 22 | 6,10-Dodecadien-1-yn-3-ol, 3,7,11-trimethyl- | SU | GCMS | 2387-68-0 | 793 | 220.182 | 41, 67, 95, 138 | GCMS | The constituents were identified after comparison with NIST library of the GCMS system. |
| 23 | 2,7-dimethyl-3-Octen-5-yne, (Z)- | KAv | GCMS | 28935-76-4 | 869 | 136.125 | 93, 121, 136 | GCMS | Compounds were identified by comparing with the NIST library. |
| 24 | 1,3,5-Cycloheptatriene | AS, PR, SA, SU, KAr, KAV | GCMS | 544-25-2 | 955 (AS)  958 (PR)  951 (SA)  956 (KAr)  950 (KAv) | 92.0626 | 65, 91 | GCMS | The spectra of the compound were matched with NIST library. The identity of the compound was determined by the percent similarity of the spectra. |
| 25 | Bicyclo[3.1.0]hexane, 4-methyl-1-(1-methylethyl)-, didehydro deriv. | DR, PR, SA, SU | GCMS | 58037-87-9 | 839 (DR), 891 (PR),  875 (SA)  837 (SU) | 136.1252 | 41, 77, 93 | GCMS | The library searches and spectral matching of compounds were conducted on the NIST 107 MS database and on the basis of RI |
| 26 | Bicyclo[3.2.1]oct-2-ene, 3-methyl-4-methylene- | DR, SU, KAv | GCMS | 49826-53-1 | 832 (DR)  869 (SU)  861 (KAv) | 134.1096 | 91, 105, 134 | GCMS | Compounds were identified by comparing with NIST library. |
| 27 | Oxirane, 2-(hexyn-1-yl)-3-methoxymethylene- | DR, KAr, KAv | GCMS | PubChem CID:  5367383 | 728 (DR)  719(KAr)  733 (KAv) | 166.0994 | 79, 110 | GCMS | The fragmentation pattern of mass spectra were compared with NIST database. |
| 28 | Bergamotol, Z-α-trans- | AS, SA | GCMS | 88034-74-6 | 828 (AS)  822 (SA) | 220.182 | 91,93, 119, 187 | GCMS | Identification of each compound was done by equating the retention time and fragmentation pattern with the spectral data obtained from NIST2011 and Wiley-10^th^ edition mass spectral libraries and literature survey. |
| 29 | (1,3-Dimethyl-2-methylene-cyclopentyl)-methanol | AS, DR, SA, SU, KAv | GCMS | PubChem CID:  572858 | (AS)  764 (DR)  760 (SA)  782 (SU)  709 (KAv) | 140.1201 | 67,77,94, 109 | GCMS | The identification of the components was based on comparison of their relative retention times and mass spectra with those of GCMS library data. |
| 30 | 12-Oxabicyclo[9.1.0]dodeca-3,7-diene, 1,5,5,8-tetramethyl-, [1R-(1R*,3E,7E,11R*)]- | DR, KAr | GCMS | 19888-34-7 | 782 (DR)  775 (KAr) | 220.1827 | 67,96, 109,138 | GCMS | Compared the compounds with NIST library. |
| 31 | Isolongifolene, 4,5,9,10-dehydro- | AS, DR, SA, SU, KAr | GCMS | 156747-45-4 | 830 (AS)  841 (DR)  843 (SA)  869 (SU)  798 (KAr) | 200.1565 | 77, 91, 143, 157, 185 | GCMS | Compounds were identified by comparing with Kovat’s retention indices and mass spectra was compared with literature and NIST library. |
| 32 | Z,Z,Z-4,6,9-Nonadecatriene | DR, KAv | GCMS | PubChem CID:  5362863 | 790 (DR)  838 (KAv) | 262.2661 | 79,93 | GCMS | The NIST version 2.0 library database was used for identifying the chemical components. |
| 33 | 6-(p-Tolyl)-2-methyl-2-heptenol | AS, SU, KAv | GCMS | 39599-18-3 | 721 (AS)  784 (SU)  734 (KAv) | 218.167 | 91,119, 202 | GCMS | Compounds were identified by matching with NIST database |
| 34 | 6-Tridecen-4-yne, (Z)- | DR, PR, SU | GCMS | 74744-45-9 | 717 (DR)  735 (PR)  704 (SU) | 178.1722 | 43,79,94 | GCMS | Compounds were identified by peak matching with library search, published mass spectra and by analysis of authentic references. |
| 35 | 1,4-Cyclohexadiene, 1-methyl- | KAr, KAv | GCMS | 4313-57-9 | 729 (KAv) 896(KAr) | 94.0783 | 55,79,94 | SPME-GCMS  (Slid phase micro extraction-GCMS) | Each compound was represented by a single selective ion fragment and then subjected to a tentative identification using the NIST mass spectral library. |
