# Supplementary material for: Variable Secondary Metabolite Profiles Across Cultivars of Curcuma longa L. and C. aromatica Salisb
Source: Front Pharmacol. 2021 Jun 30;12:659546. doi: 10.3389/fphar.2021.659546 (PMC8278146; doi:10.3389/fphar.2021.659546)
Supplement: Supplementary file 6 [file Table4.docx]

**Supplementary Table S4.** Identification parameters of first-time reported compounds by LC-MS in the rhizome extracts of genus *Curcuma* along with the methods used in related previous literature.

| **Sl. nos.** | **Compound** | **Cultivar** | **Identification of the compounds done in present study** | | | **Identification of the compounds done in previous literature** | |
| --- | --- | --- | --- | --- | --- | --- | --- |
|  |  |  | **Technique used** | **Mass (m/z)** | **Mass fragmentation pattern (m/z)** | **Technique used** | **Compound identification method** |
| 1 | Kaempferol-3,7-O-dimethyl ether | AS | LCMS | 313.0721 M-H^-^ | 108,123, 152,153,  167 | TLC (Nikolova, 2006) and Electronspray ionization tandem mass spectrometry (DeSouza et al., 2010) | Compounds identified matching TLC spot with the standard (Nikolova, 2006);  ESI-MS/MS led to the identification of compound on the basis of its molecular mass and fragmentation observed in the MS/MS spectrum of the flavonoids selected from the first order ESI-MS spectrum of the flavonoid rich fraction (DeSouza et al., 2010) |
| 2 | 5,7,8-Trihydroxy-2′,5′-dimethoxy-3′,4′-methylene dioxyisoflavanone | AS, DR | LCMS | 377.1059  M-H^-^ | 101,102, 113,119, 161, 163,228,336 | UV, IR, MS, 1D and 2D NMR | Structure was elucidated by interpretation of UV, IR, MS, 1D and 2D NMR data. |
| 3 | Chavicol | AS, SU | LCMS | 135.0794 M+H^+^ | 102,115,  116 | DART-MS | Exact mass calibration was accomplished by including a mass spectrum of neat polyethylene (PEG) glycol (1:1 mixture PEG 200 and PEG 600) in data file. *m-*Nitrobenzyle alcohol was also used for calibration. |
| 4 | Kaempferol-3-O-rutinoside-7-O-glucoside | PR, SA | LCMS | 755.2655  M- H^-^ | 135,161,175,176,191,439,579,755,756 | ^1^H-NMR and multivariate analysis | Compound was identified by analyzing the spectrum using 2D experiments, combined information gathered from COSY and HOHAHA spectra and the use of a library of ^1^H spectra of reference compounds. |
