# Supplementary material for: Variable Secondary Metabolite Profiles Across Cultivars of Curcuma longa L. and C. aromatica Salisb
Source: Front Pharmacol. 2021 Jun 30;12:659546. doi: 10.3389/fphar.2021.659546 (PMC8278146; doi:10.3389/fphar.2021.659546)
Supplement: Supplementary file 7 [file Table5.docx]

**Supplementary Table** S**5**. The list of 80 compounds identified by GCMS analysis.

| **Sl. Nos.** | **Name of compound** | **Class of compound** |
| --- | --- | --- |
| **Compounds specific for each cultivar** | | |
| 1 | 1,2-Cyclohexanediol, 1-methyl-4-(1-methylethyl)- | monoterpenoid |
| 2 | trans, trans-Octa-2,4-dienyl acetate | dienyl acetate |
| 3 | Phenol, 2-methoxy-3-(2- propenyl)- | phenolic monoterpenoid |
| 4 | 3-Isopropyl-4-methyl-1-pentyn-3-ol | alcohol constituent |
| 5 | 5,9-Tetradecadiyne | unsaturated hydrocarbon |
| 6 | Naphthalene, 5-butyl-1,2,3,4-tetrahydro- | tetralin Type |
| 7 | Santolina alcohol | tertiary alcohol |
| 8 | 2-Pentanone, 4-mercapto-4-methyl- | ketone |
| 9 | 8-Methylene-3-oxatricyclo[5.2.0.0(2,4)]nonane | hydrocarbon |
| 10 | 7-Tetracyclo[6.2.1.0(3.8)0(3.9)]undecanol, 4,4,11,11 tetramethyl- | sesquiterpene alcohol |
| 11 | Bicyclo[2.2.1]hept-2-ene, 2,3-dimethyl- | cyclic hydrocarbon |
| 12 | 1H-3a,7-Methanoazulene, 2,3,4,7,8,8a-hexahydro-3,6,8,8-tetramethyl-, [3R-(3à,3aá,7á,8aà)]- | sesquiterpene |
| 13 | Cholesta-8,24-dien-3-ol, 4-methyl-, (3á,4à)- | triterpenoid |
| 14 | 4-Ethylphenethylamine | amine |
| 15 | Cyclohexanol, 2-methyl-5-(1-methylethenyl)- | monoterpenoid |
| 16 | Cyclohexane, 1,2-dimethyl-3,5-bis(1-methylethenyl)- | monoterpenoid |
| 17 | 5,8,11,14-Eicosatetraenoic acid, phenylmethyl ester, (all-Z)- | ester |
| 18 | 11-Dodecen-2-one | ketone |
| 19 | E-11-Tetradecenoic acid | fatty acid |
| 20 | 2-Nonen-4-yn-1-ol, (Z)- | alcohol |
| 21 | 3-Cyclohexen-1-one, 3,5,5-trimethyl- | cyclohexenone |
| 22 | 6,10-Dodecadien-1-yn-3-ol, 3,7,11-trimethyl- | sesquiterpenoid |
| 23 | 3-Octen-5-yne, 2,7-dimethyl-, (Z)- | monoterpene |
| 24 | Aromadendrene | hydrocarbon |
| 25 | Isoborneol | monoterpenoid |
| 26 | β-Elemene | sesquiterpene |
| 27 | α-Santalene | sesquiterpene |
| 28 | 2-Tridecanone | ketone |
| 29 | Nonanoic acid | fatty acid |
| 30 | Eucalyptol | monoterpene |
| 31 | Carvacrol | monoterpene |
| 32 | endo-Borneol | monoterpene |
| 33 | 1,3,5-Cycloheptatriene, 3,7,7-trimethyl- | cyclic hydrocarbon |
| 34 | p-Cymen-8-ol | monoterpenoid |
| 35 | Camphor | terpenoid ketone |
| 36 | α-Bisabolol | sesquiterpenoid |
| 37 | α-Elemenone | sesquiterpene |
| 38 | Caryophyllene oxide | sesquiterpenoid oxide |
| 39 | Citral | monoterpene |
| 40 | Neoisolongifolene, 8,9-dehydro- | bicyclic hydrocarbon |
| 41 | Sabinene hydrate | monoterpene |
| **Compounds found in more than one cultivar** | | |
| 1 | 1,3,5-Cycloheptatriene | closed ring organic compound |
| 2 | Bicyclo[3.1.0]hexane, 4-methyl-1-(1-methylethyl)-, didehydro deriv. | monoterpene |
| 3 | Bicyclo[3.2.1]oct-2-ene, 3-methyl-4-methylene- | monoterpene |
| 4 | Oxirane, 2-(hexyn-1-yl)-3-methoxymethylene- | cyclic ether and epoxide |
| 5 | Bergamotol, Z-α-trans- | sesquiterpene alcohol |
| 6 | (1,3-Dimethyl-2-methylene-cyclopentyl)-methanol | alcohol |
| 7 | 12-Oxabicyclo[9.1.0]dodeca-3,7-diene, 1,5,5,8-tetramethyl-, [1R-(1R*,3E,7E,11R*)]- | epoxide |
| 8 | Isolongifolene, 4,5,9,10-dehydro- | polycyclic hydrocarbon |
| 9 | Z,Z,Z-4,6,9-Nonadecatriene | hydrocarbon |
| 10 | 6-(p-Tolyl)-2-methyl-2-heptenol | aromatic alcohol |
| 11 | 6-Tridecen-4-yne, (Z)- | hydrocarbon |
| 12 | 1,4-Cyclohexadiene, 1-methyl- | aromatic alcohol |
| 13 | Camphene | monoterpene |
| 14 | α-Phellandrene | monoterpene |
| 15 | Limonene | monoterpene |
| 16 | α-Terpineol | monoterpenoid |
| 17 | β-Sesquiphellandren | sesquiterpene |
| 18 | Nerolidol | sesquiterpinol |
| 19 | Bicyclo[4.1.0]hept-2-ene, 3,7,7-trimethyl- | monoterpene |
| 20 | α-Terpinene | monoterpene |
| 21 | cis-Ocimene | monoterpene |
| 22 | γ-Terpinene | monoterpene |
| 23 | Linalool | alcohol |
| 24 | Terpinene-4-ol | monoterpene |
| 25 | cis-α-Bisabolene | sesquiterpene |
| 26 | Ar-Tumerone | sesquiterpene |
| **Compounds present in all 7 cultivars (common compounds)** | | |
| 1 | α-Thujene | monoterpenoid |
| 2 | 1s-α-Pinene | monoterpenoid |
| 3 | Sabinene | monoterpenoid |
| 4 | β or m-Cymene | aromatic hydrocarbon |
| 5 | Terpinolene | monoterpenoid |
| 6 | trans-α-Bergamotene | sesquiterpene |
| 7 | α-Caryophyllene | sesquiterpene |
| 8 | trans-β-Farnesene | sesquiterpene |
| 9 | Ar-Curcumene | sesquiterpene |
| 10 | α-Zingiberene | sesquiterpene |
| 11 | Tumerone | sesquiterpene |
| 12 | Curlone | sesquiterpene |
| 13 | 2-Heptadecanone | ketone |
| **Total** | 41+26+13=80 | |
