# Supplementary material for: Variable Secondary Metabolite Profiles Across Cultivars of Curcuma longa L. and C. aromatica Salisb
Source: Front Pharmacol. 2021 Jun 30;12:659546. doi: 10.3389/fphar.2021.659546 (PMC8278146; doi:10.3389/fphar.2021.659546)
Supplement: Supplementary file 8 [file Table6.docx]

**Supplementary Table S6**. The list of 62 compounds identified by LCMS analysis.

| **Sl. Nos.** | **Name of compound** | **Class of compound** |
| --- | --- | --- |
| **Compounds specific for each cultivar** | | |
| 1 | Kaempferol-3,7-O-dimethyl ether | flavonoid |
| 2 | Luteolin-7-O-glucoside | flavonoid |
| 3 | 1,7-Bis(4-hydroxy-3,5-dimethoxyphenyl)-1,6-heptadiene-3,5-dione | diarylheptanoid |
| 4 | 1,7-Bis(3,4-dimethoxyphenyl)-1,6-heptadiene-3,5-dione | diarylheptanoid |
| 5 | (6S)-2-Methyl-6-[(1R,5S)-(4-methene-5-hydroxyl-2-cyclohexen)-2-hepten-4-one | bisabolane |
| 6 | 1,7-Bis(4-hydroxyphenyl)-3,5-heptanediol | diarylheptanoid |
| 7 | 1,7-Bis(3,5-diethyl-4-hydroxyphenyl)-1,6-heptadiene-3,5-dione | aromatic |
| 8 | 1-(4-Hydroxy-3-methoxyphenyl)-5-(4-hydroxyphenyl)-1,4-pentadiene-3-one | diarylheptanoid |
| 9 | (-)-(12E,2S,3S,4R, 5R,6R, 9S,11S, 15R)-3,15-Dibenzoyloxy-5,6-epoxylathyr-12-en-14-one | diterpenoid |
| 10 | 5’-Methoxycurcumin | diarylheptanoid |
| 11 | Methyl-7-methoxycoumarin,4- | coumarin |
| 12 | Hydroferulic acid | phenolic acid |
| 13 | 1,2,3,4-Tetraphenylbutane-2,3-diol | aliphatic diol |
| 14 | 4-Hepten-3-one, 5-hydroxy-1,7-bis(4-hydroxy phenyl)- | diarylheptanoid |
| 15 | 5,7-Dihydroxy-2-(4-hydroxyphenyl)-chroman-4-one | phenolic acids |
| 16 | Tetradecanoic acid/myristic acid | fatty acid |
| 17 | 1-(4-Hydroxy-3-methoxyphenyl)-7-(4-hydroxy-3,5-dimethoxypheny)-4,6-heptadiene-3-one | diarylheptanoid |
| 18 | Tumerone | bisabolane sesquiterpene |
| 19 | 4-Methylene-5-hydroxybisabola-2,10-diene-9-one | sesquiterpene |
| 20 | 1,7-Bis(3,4-dimethoxyphenyl)-4,4-dimethyl-1,6-heptadiene-3,5-dione | diarylheptanoid |
| 21 | 25-Benzylpentacyclo-(22.3.1.0.)-octacosa-1(27),3 (8),4,6,10(15),11,13,17(22), 18,20, 24(28), 25-dodecaen | cyclic diarylheptanoid |
| 22 | Palmitic acid | fatty acid |
| 23 | Stearic acid | fatty acid |
| **Compounds found in more than one cultivar** | | |
| 1 | 5,7,8-Trihydroxy-2′,5′-dimethoxy-3′,4′-methylene dioxyisoflavanone | flavonoid |
| 2 | Chavicol | terpenoid |
| 3 | Kaempferol-3-O-rutinoside-7-O-glucoside | flavonoid |
| 4 | Kaempferol-3-rhamnoside | flavonoid |
| 5 | 3-Acetyl coumarin | Coumarin |
| 6 | Turmeronol | bisabolane sesquiterpene |
| 7 | 7-(3,4-Dihydroxyphenyl)-5-hydroxy-1-phenyl-(1E)-1-heptene | diarylheptanoid |
| 8 | 1,7-Diphenyl-1,6-heptadiene-3,5-dione | diarylheptanoid |
| 9 | 1-Hepten-3-one, 5-hydroxy-1,7-bis(3,4-dihydroxyphenyl)- | diarylheptanoid |
| 10 | 4-(*p*-Hydroxyphenyl)-3-buten-2-one | flavonoid |
| 11 | 5-Hydroxy-7-(4-hydroxyphenyl)-1-phenyl-(1E)-1-heptene | diarylheptanoid |
| 12 | 1-(4-Hydroxy-3-methoxyphenyl)-7-(4-hydroxy-3,5-dimethoxypheny)-4,6-heptadiene-3-one | diarylheptanoid |
| 13 | 1,5-Bis(3,4-methylenedioxyphenyl)-1,4-pentadien-3-one | diarylheptanoid |
| 14 | 1-Hydroxy-1-(3,4-dihydroxyphenyl)-7-(4-hydroxy-3-methoxyphenyl)-6-hepten-3,5-dione | diarylheptanoid |
| 15 | 1,7-Bis(4-hydroxyphenyl)-1-heptene-3,5-dione | diarylheptanoid |
| 16 | 1,7-Bis(4-hydroxyphenyl)-1,4,6-heptatrien-3-one | diarylheptanoid |
| 17 | 7-(4-Hydroxy-3-methoxyphenyl)-1-(4-hydroxyphenyl)-4,6-heptadien-3-one | diarylheptanoid |
| 18 | Coumaran | coumarin |
| 19 | 5,7-Dihydroxy-4-methylcoumarin | coumarin |
| 20 | 1,7-Bis(3,4,5-trimethoxyphenyl)-l,6-heptadiene-3,5-dione | diarylheptanoid |
| 21 | Curlone | bisabolane sesquiterpene |
| 22 | Hydrocinnamic acid | phenolic acid |
| 23 | Curcumenol | sesquiterpene |
| 24 | Oleic acid | fatty acid |
| **Compounds common for all seven cultivars** | | |
| 1 | 1,5-Bis(4-hydroxy-3-methoxyphenyl)-1,4-pentadien-3-one | diarylheptanoid |
| 2 | Ar-Turmerone | bisabolane sesquiterpene |
| 3 | Tetrahydroxybisdemethoxycurcumin | diarylheptanoid |
| 4 | Tetrahydrodemethoxycurcumin | diarylheptanoid |
| 5 | 1-(4-Hydroxyphenyl)-7-(4-hydroxy-3-methoxyphenyl)-1,4,6-heptatrien-3-one | diarylheptanoid |
| 6 | 1,7-Bis(4-hydroxy-3-methoxyphenyl)-1,4,6-heptatrien-3-one | diarylheptanoid |
| 7 | Tetrahydroxycurcumin | diarylheptanoid |
| 8 | 1-(4-Hydroxy-3-methoxyphenyl)-7-(4-hydroxy-3,5-dimethoxyphenyl)-1,4,6-heptatrien-3-one | diarylheptanoid |
| 9 | 1,6-Heptadiene-3,5-dione, 1-(3,4-dihydroxyphenyl)-7-(4-hydroxy phenyl)- | diarylheptanoid |
| 10 | 1-(3,4-Dihydroxyphenyl)-7-(4-hydroxy-3-methoxyphenyl)-hepta-1,6-diene-3,5-dione | diarylheptanoid |
| 11 | Bisdemthoxycurcumin | diarylheptanoid |
| 12 | Demethoxycurcumin | diarylheptanoid |
| 13 | Dihydrocurcumin | diarylheptanoid |
| 14 | Curcumin | diarylheptanoid |
| 15 | 1-Heptene-3,5-dione, 1,7-bis-(4-hydroxy-3-methoxyphenyl)- | diarylheptanoid |
| **Total** | 23+24+15=62 | |
